# Association Mapping of Insecticide Resistance in Wild *Anopheles gambiae* Populations: Major Variants Identified in a Low-Linkage Disequilbrium Genome

**DOI:** 10.1371/journal.pone.0013140

**Published:** 2010-10-01

**Authors:** David Weetman, Craig S. Wilding, Keith Steen, John C. Morgan, Frédéric Simard, Martin J. Donnelly

**Affiliations:** 1 Vector Group, Liverpool School of Tropical Medicine, Pembroke Place, Liverpool, United Kingdom; 2 Laboratoire de Lutte contre les Insectes Nuisibles (LIN), Institut de Recherche pour le Développement (IRD), UR016, Montpellier, France; The University of Hong Kong, China

## Abstract

**Background:**

Association studies are a promising way to uncover the genetic basis of complex traits in wild populations. Data on population stratification, linkage disequilibrium and distribution of variant effect-sizes for different trait-types are required to predict study success but are lacking for most taxa. We quantified and investigated the impacts of these key variables in a large-scale association study of a strongly selected trait of medical importance: pyrethroid resistance in the African malaria vector *Anopheles gambiae*.

**Methodology/Principal Findings:**

We genotyped ≈1500 resistance-phenotyped wild mosquitoes from Ghana and Cameroon using a 1536-SNP array enriched for candidate insecticide resistance gene SNPs. Three factors greatly impacted study power. (1) Population stratification, which was attributable to co-occurrence of molecular forms (M and S), and cryptic within-form stratification necessitating both a partitioned analysis and genomic control. (2) All SNPs of substantial effect (odds ratio, OR>2) were rare (minor allele frequency, MAF<0.05). (3) Linkage disequilibrium (LD) was very low throughout most of the genome. Nevertheless, locally high LD, consistent with a recent selective sweep, and uniformly high ORs in each subsample facilitated significant direct and indirect detection of the known insecticide target site mutation *kdr L1014F* (OR≈6; P<10^−6^), but with resistance level modified by local haplotypic background.

**Conclusion:**

Primarily as a result of very low LD in wild *A. Gambiae,* LD-based association mapping is challenging, but is feasible at least for major effect variants, especially where LD is enhanced by selective sweeps. Such variants will be of greatest importance for predictive diagnostic screening.

## Introduction

*Anopheles gambiae* is the most important vector of malaria, which endangers almost half the world's population and causes nearly a million deaths annually, most in children under five [1]. Insecticide-based vector control is a proven method for disease control [2] and a key component of integrated malaria control programmes [1], [3] but is threatened by resistance to all insecticides licensed for public health use by the World Health Organization (WHO) [4]. Identification of the loci and polymorphisms in the *A. gambiae* genome that play a major role in insecticide resistance could greatly enhance malaria control efforts by providing predictive resistance-diagnostics and information for design of new insecticides [3]. Several types of mechanisms might produce resistant phenotypes in *Anopheles* mosquitoes, but available evidence suggests that the most important are alterations to the target site of the insecticide and metabolic resistance via altered expression of detoxification genes [5]. The four classes of WHO-approved insecticides share only two target sites, a voltage-gated sodium channel and an acetylcholinesterase gene. Resistance-associated mutations in both genes have been found in *A. gambiae* [6], [7], [8] and at least one of the two known sodium channel (‘knockdown resistance’, *kdr*) mutations has a wide distribution throughout West and Central Africa, and often occurs at high frequencies [9]. Microarray studies comparing laboratory colonies or field isolates differing in susceptibility to insecticide have detected resistance-associated overexpression of multiple detoxification genes, with cytochrome P450 monooxygenases in particular arising consistently [10], [11], [12]. In *A. gambiae*, DNA polymorphisms associated with metabolic resistance in field samples have yet to be discovered, and the relative importance of target site resistance and metabolic detoxification mechanisms in natural populations is unclear [13]. Comprehensive candidate gene screens of natural mosquito populations represent a logical and promising next step to identify a broader spectrum of genetic variants important in insecticide resistance and could also pave the way for subsequent genomewide association (GWA) studies.

Sequencing of the *A. gambiae* genome [14], and subsequent re-sequencing [15], [16], [17] has identified sufficient single nucleotide polymorphisms (SNPs) to make GWA studies of medically-relevant phenotypes in natural populations feasible. However, GWA studies have not yet been performed in insects, and it is unclear whether the rigorous methodologies developed to identify the genetic variants underlying common human diseases in European populations [18] will prove successful. Indeed, these methodologies, which comprise of genotyping using tag-SNP arrays, stringent statistical criteria for association in single populations, and replication of results in large multi-population studies appear difficult to apply in African human populations because of much lower linkage disequilibrium (LD) and greater population structure [19], [20]. LD is a key variable determining the power of association studies [21]. Though there are few data on LD in insect disease vectors the large population sizes expected suggest it may be far less extensive than in humans and the domesticated species which have been the subject of most LD mapping studies to date. Moreover, the population genetics and genomics of mosquitoes and humans differ markedly. For example *A. gambiae* exhibits variably-permeable species boundaries, particularly between the M and S molecular forms, which are thought to represent incipient species [22]. However, it is unclear whether differentiation between molecular forms is genomewide [23] or localised primarily to a few low-recombination regions, most notably toward the centromeres of chromosomes X, 2L and 3L [24], [25]. Similarly, large paracentric chromosomal inversions in *A. gambiae* [26] may maintain LD and differentiation over only parts of the genome, or may be a driving force in ecological population differentiation [27], [28]. Until these issues are resolved appropriate strategies to treat within-sample mixtures of both molecular and chromosomal forms in association analyses are unclear. Here we address the extent to which LD and within- and among-population structure affect association studies via the first large-scale study of the genetic architecture of insecticide resistance in wild *Anopheles* populations.

Using a custom-designed 1536-SNP array (886 SNPs scored successfully), enriched for SNPs within or around >250 candidate insecticide resistance genes, we screened *A. gambiae* sampled from sites in Ghana and Cameroon contrasting markedly in resistance level. Individuals were classified as resistant or susceptible to permethrin (a class I pyrethroid, used widely to treat bednets). In addition to identifying resistance-associated polymorphisms, our results reveal how LD and within-population structure impact study power, so highlighting critical issues for the design of future association studies of wild populations of *A. gambiae* and other taxa.

## Results

### Insecticide resistance phenotypes

**Figure 1:**
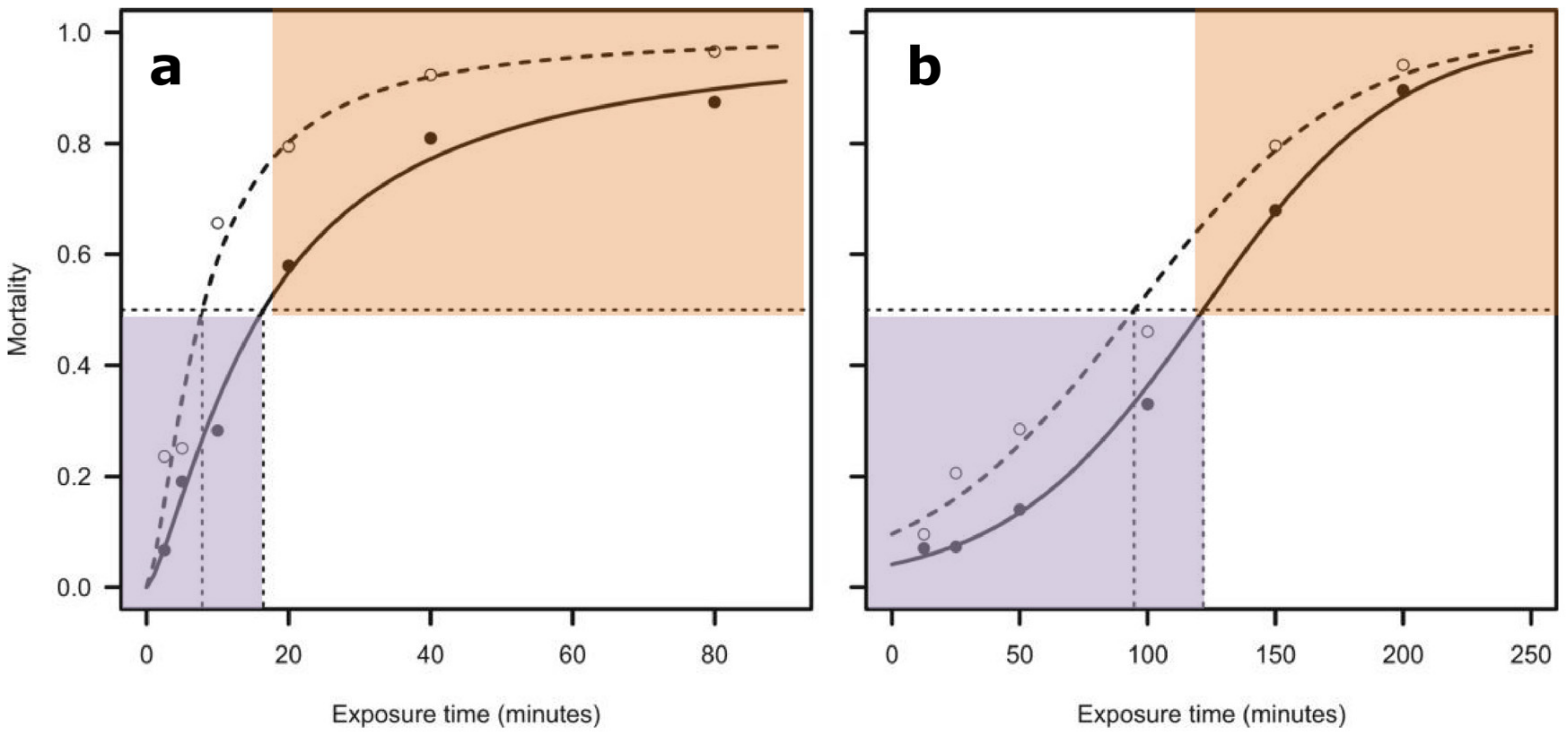
Determination of permethrin-resistance phenotypes. In (a) Cameroon and (b) Ghana the LT_50_ (time point at which dotted line meets x-axis) for females (filled markers and solid curve) and males (open markers and dashed curve) was determined and used as the phenotypic threshold with individuals failing to survive this duration of insecticide exposure classified as susceptible (purple shading) and survivors classified as resistant (orange shading). Note that males were not included in subsequent genetic analysis.

All individuals were screened for resistance to the type I pyrethroid insecticide permethrin. In Cameroon the LT_50_ for permethrin for females was only 16.8 min (Figure 1) but there was evidence of resistance in at least a proportion of the population with nearly 20% surviving at the WHO standard exposure time of 60 min. By contrast Ghanaian females were strongly resistant with around 80% survival at 60 min and an LT_50_ of 122 min. Prior to genotyping using the SNP array the *A. gambiae s.l.* species and *A. gambiae s.s.* molecular form of all samples was characterised using standard diagnostics [29], [30]. All were *A. gambiae s.s.* with a 92%:8% (M:S) division in Cameroon and 2%:98% in Ghana (with two M/S hybrids in Ghana). In both collections S molecular forms were much more permethrin-resistant than M forms (Ghana = χ^2^_1_ = 11.55, *P* = 0.0007; Cameroon χ^2^_1_ = 35.9, *P* = 2×10^−9^).

### Population structure and stratification

**Figure 2:**
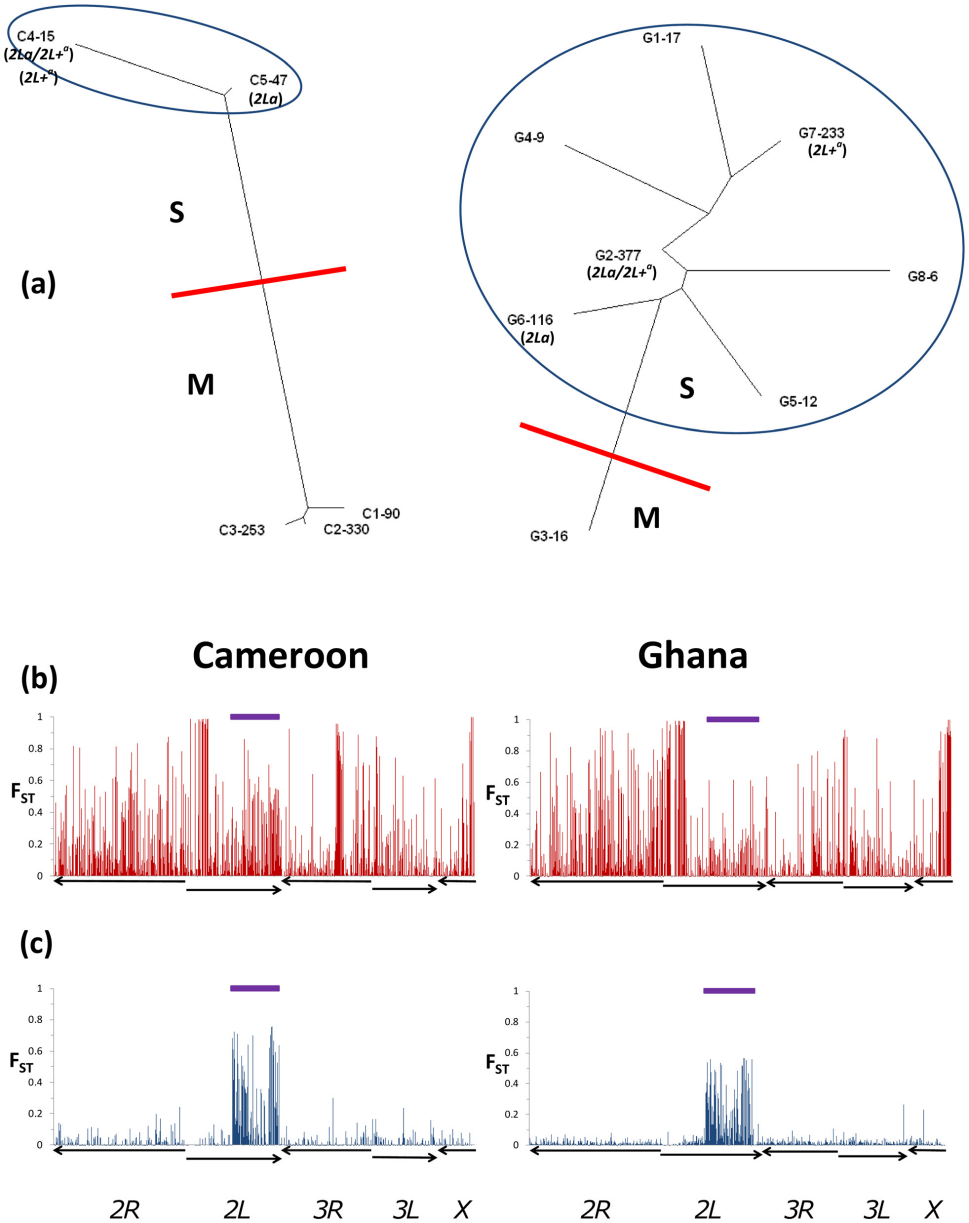
Population structure in the sample collections. (a) Genotype clusters identified are shown in neighbour-joining trees, based on the Kullback-Leibler (KL) distance, with cluster names and sizes shown (name-*N*). Red lines show demarcation between molecular forms and in (b) red bars show the corresponding F_ST_-value for each SNP. Blue circles in (a) delimit the remainder of the sample among which there was marked differentiation and in (c) blue bars the corresponding F_ST_ for each SNP. 2La karyotypes are indicated. Purple bars at the top of each plot indicates the position of the 2La inversion region. Arrows indicate chromosomes as labeled (oriented centromere-telomere).

To determine the most appropriate way to conduct association tests we sought not only to identify units of within-population structure but also the uniformity of differentiation throughout the genome between such units. We knew already that molecular forms represented a source of stratification (i.e. they were unequally distributed between the resistant and susceptible phenotypic groupings) and would require some form of correction in the analysis, but the key question was whether differentiation was localised or widespread throughout the genome. Cluster analysis using approximately 200 control (non-candidate gene) SNPs, dispersed throughout the genome (Table S1) identified marked structure within each collection (Figure 2) attributable to two major sources: differentiation between the molecular forms of *A*. *gambiae*, and between individuals possessing different inversion polymorphisms. M and S forms were highly differentiated in both the Cameroon (global F_ST_ = 0.24) and Ghana (F_ST_ = 0.17) collections. SNPs near the centromeres of chromosomes X and 2L exhibited the most extreme divergence between M and S clusters, but high differentiation was widespread throughout the genome. By contrast, differentiation among clusters within each molecular form was primarily attributable to localised divergence in the region of a large (≈20 Mb) inversion of chromosome 2L (known as 2La), being especially pronounced toward the ends of the inversion. Typing using a simple PCR diagnostic [31] revealed a perfect split among these clusters for 2La karyotypes in Ghana S forms, near perfect in Cameroon S forms (Figure 2), but no no relationship between clustering and 2La inversion karyotypes in Cameroon M forms (Table S2).

**Figure 3:**
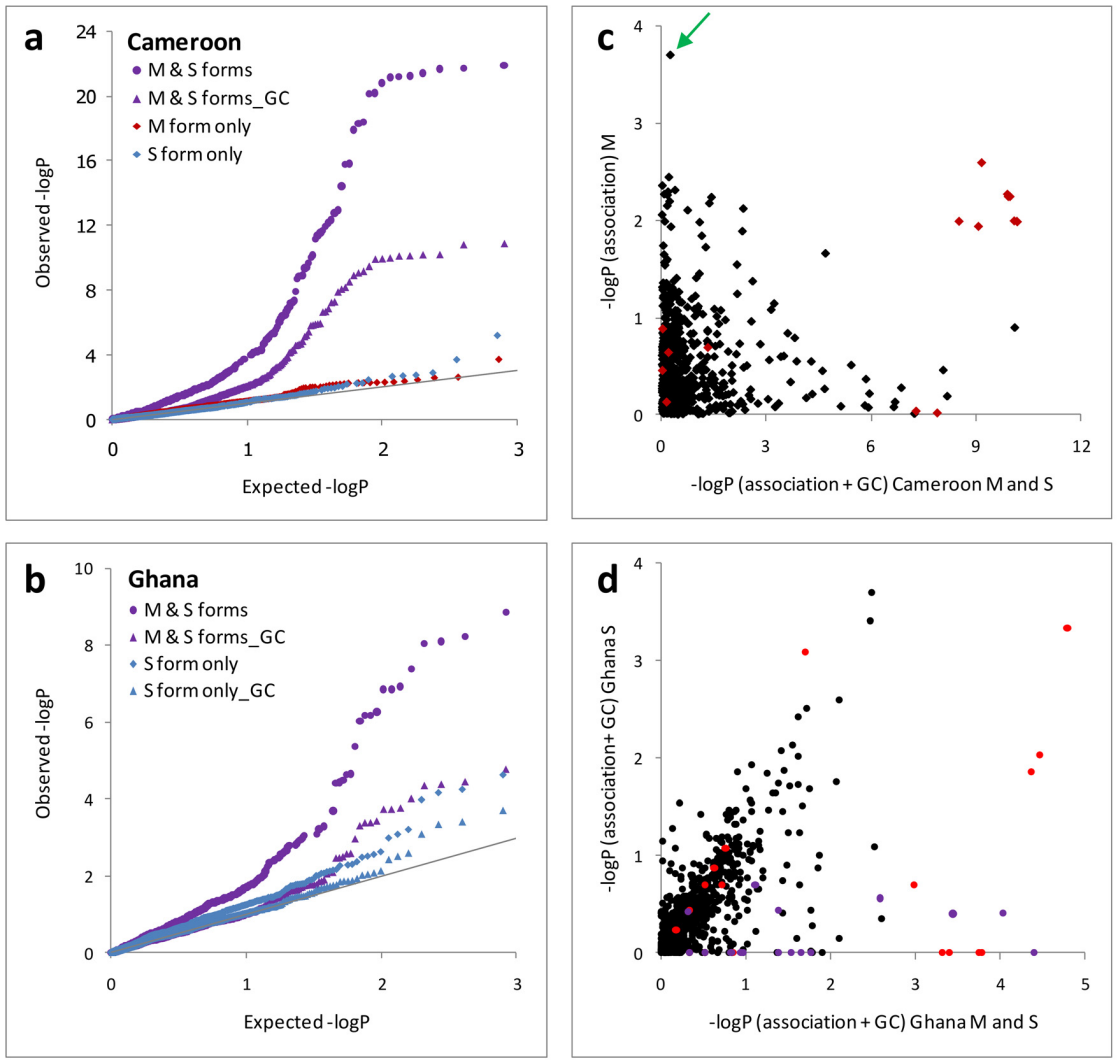
Evaluation and correction of molecular form-related stratification in association tests. (a,b) Failure to partition M and S forms leads to massive inflation of observed –logP values relative to their expected distribution (solid line). Within Ghanaian S forms genomic control (GC) is required to align results for the majority of SNPs to their expectation. (c,d) False positives (high x-axis, low y-axis values) are common in unpartitioned datasets and primarily attributable to SNPs in the sodium channel (red) and 2L or X speciation island regions (defined conservatively as <2Mb and >20Mb respectively [24] (purple) in the Ghana dataset, though in Cameroon, SNPs in other areas (black) appear as false positives, which also shows a possible false negative (green arrow).

Observed *vs.* expected association probability plots were used to visually assess evidence for population stratification [32], and the genomic control (GC) statistic λ (defined in Materials and methods) provided a quantitative measure of inflation of the median χ^2^_1_ value for control SNPs relative to that expected under a theoretical χ^2^_1_ distribution. In both Cameroon and Ghana, when M and S forms were analysed together, most SNPs showed much higher than expected –logP values (Figure 3a,b), indicative of stratification-related inflation and a very high false positive rate [32]. We assessed two possible methods of correction for stratification: a partitioned analysis (M and S analysed separately in Cameroon; and simply exclusion of M forms from Ghana owing to their rarity), and statistical correction of molecular form-related stratification by GC. For the Cameroon data, despite the application of a huge value of λ (31.78) stratification could not be removed from the data by GC (Figure 3a) and to avoid both false positives and negatives (Figure 3c) a partitioned analysis of M and S was essential. Once forms were separated, GC was not required for either the Cameroon M or S form dataset with λ<1 in each case, indicating that stratification was effectively removed by simply partitioning the forms. For the combined (M and S form) analysis in Ghana, GC (using λ = 1.98). produced much improved alignment of the majority of statistics to their expectations (Figure 3b). However, examination of the SNPs involved showed this analysis to be unsafe, with clear evidence of false positives (Figure 3d). Even for S forms alone however, an unacceptable [19], [33] level of inflation remained, with λ much greater than its expectation of unity (λ = 1.30). This inflation was not related to the structure reflected by the 2La inversion or the more complex clustering in Ghana in general because λ was identical (1.30) if recalculated using only samples from the single largest 2La karyotype homozygote cluster (G7 in Figure 2).

To investigate further the stratification in the Ghanaian sample we removed the two major sources of structure - i.e. M forms and all control SNPs within the 2La inversion - and repeated the clustering analysis (Figure S1). A similar clustering architecture was obtained with three major clusters (approximately 90% of all S forms) and multiple minor ones, though now differentiation among the major clusters was localised to SNPs toward the centromere of chromosome 3L and also in an inversion region of chromosome 2R (2Rb). Even though differentiation among the new major clusters was very low (global F_ST_ = 0.005), stratification was still evident (λ = 1.19; calculated for the samples within the major clusters). Therefore, cluster-based analysis was not capturing the units of evidently cryptic structure that were the source of stratification in Ghanaian S forms. As a consequence, we applied genomic control to the whole S form dataset by dividing association test χ^2^ values by λ = 1.30. This proved to be a successful strategy with general convergence between observed and expected values for the great majority of SNPs (Figure 3b). In summary, even with the application of GC, molecular forms could not be analysed together in either collection, but GC was an effective strategy for correcting cryptic residual within-form stratification.

### Linkage disequilibrium

**Figure 4:**
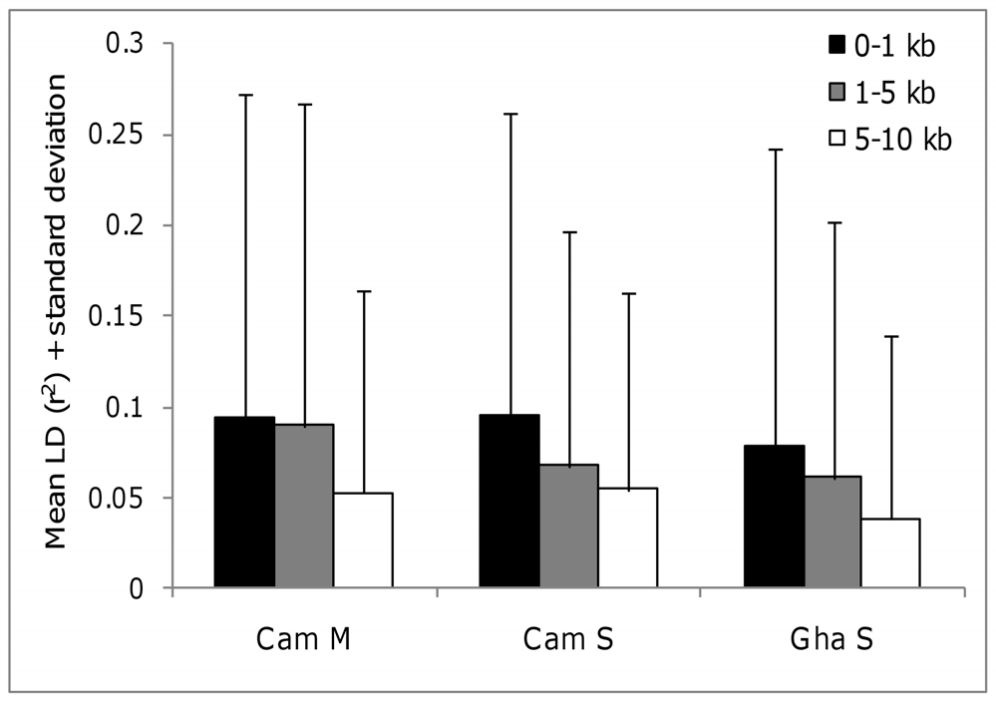
Average fine-scale linkage disequilibrium estimates.

Based on the cluster analysis results and sample sizes available, patterns of LD were assessed separately within the M and S forms in Cameroon and the S forms in Ghana (Figure S2). Some areas showed marked LD, most notably the 2La inversion region in both of the S form clusters, especially for SNPs toward the breakpoints, which were in very strong LD with one another. LD was also pronounced around the 2L voltage-gated sodium channel region in Cameroon M and Ghana S, and to a lesser extent in Cameroon S. Moderate levels of LD on chromosome 3L were found consistently in each collection extending up to approximately 6 Mb from the centromere, though were less clear in the Cameroon S subsample where the much smaller sample size yielded greater sampling error in LD estimates. Beyond these regions LD was high in only small areas or in sporadic pairwise comparisons among SNPs. Indeed, mean fine-scale r^2^ values were low even for SNPs separated by hundreds of bases (Figure 4). Since the capacity to detect the same phenotype-genotype associations across populations will depend on the consistency of LD patterns among SNPs we evaluated correlations in pairwise LD between sample sets (Table 1). For fine-scaled LD estimates (≤10 kb) correlation coefficients were low between the Cameroon M and either S form sample, but much higher between the Cameroon S and Ghana S forms, particularly for chromosomes 2L and 3L where there were extended tracts of LD in inversion and centromere-proximal regions respectively (Figure S2). Very similar results in comparisons between Ghana or Cameroon S forms and a sample of approximately 220 S forms from Uganda genotyped using the same array (mean r = 0.68 and 0.60, respectively; data not shown) suggest that, at least within the S molecular form and at a fine genomic scale, reasonable cross-population correlation in LD can be observed.

**Table 1:** Pearson correlations between populations for pairwise LD estimates (r^2^) for SNPs separated by ≤10 kb.

Sample 1	Cam M	Cam M	Cam S	
Sample 2	Cam S	Gha S	Gha S	N
2R	0.35	0.45	0.67	224
2L	0.33	0.42	0.80	143
3R	0.34	0.34	0.64	170
3L	0.31	0.34	0.94	145
X	−0.06	0.26	−0.09	62
Mean*	0.30	0.38	0.68	

*weighted by number of comparisons per chromosome (N).

### Association analysis

**Figure 5:**
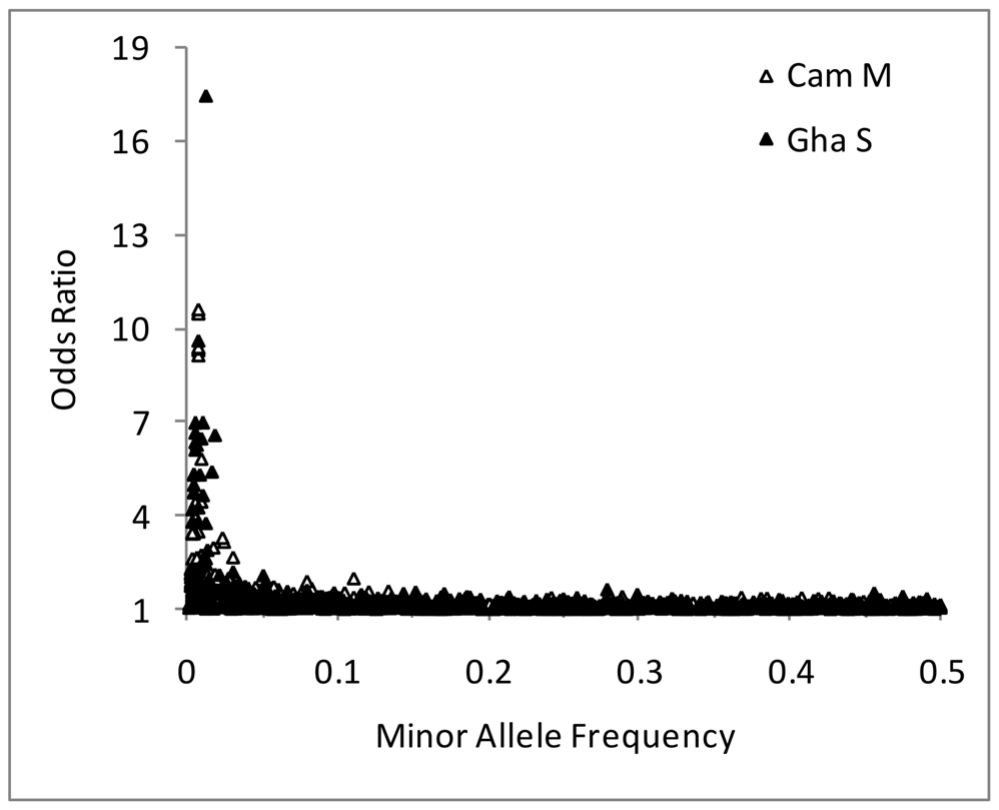
Relationship between effect size and polymorphism. Effect size is measured by odds ratio, and polymorphism by minor allele frequency in the Cameroon M and Ghana S collections. ORs are liable to inflation because of the much smaller-size in the Cameroon S collection and are not shown.

**Figure 6:**
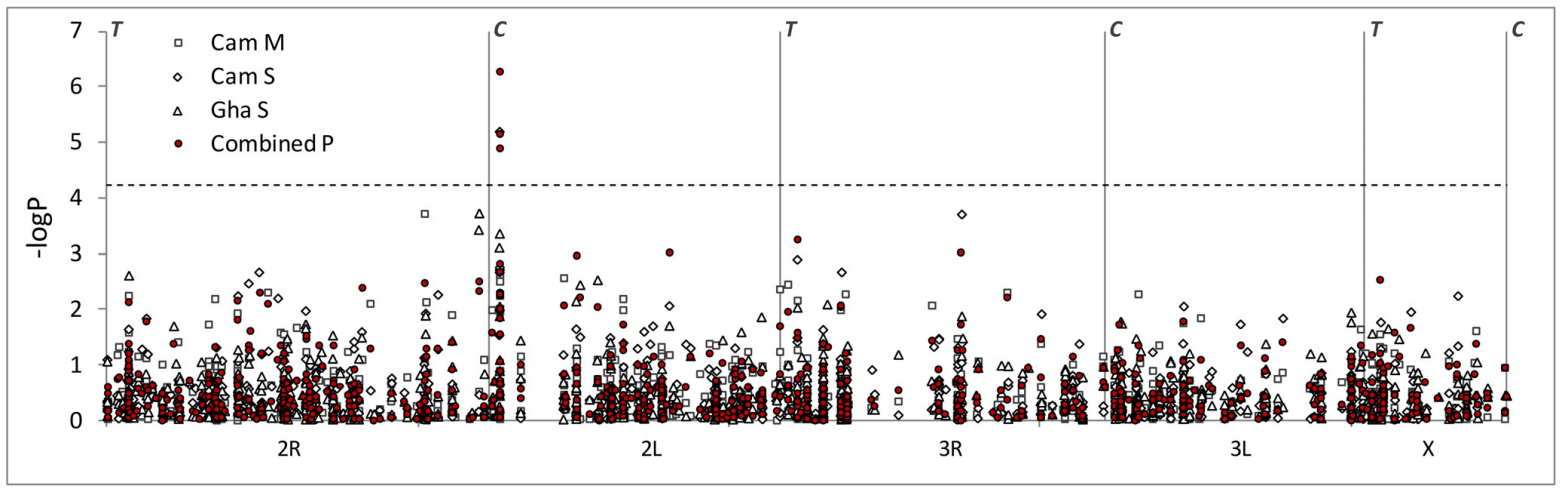
Association test results for each subsample and for P-values combined across populations (Fisher's method). The x-axis is a linear physical scale across each chromosome, with centromeres and telomeres denoted by C and T, respectively. The dashed horizontal line shows the P-value level adjusted for multiple testing by Bonferroni correction of the nominal critical α = 0.05.

**Figure 7:**
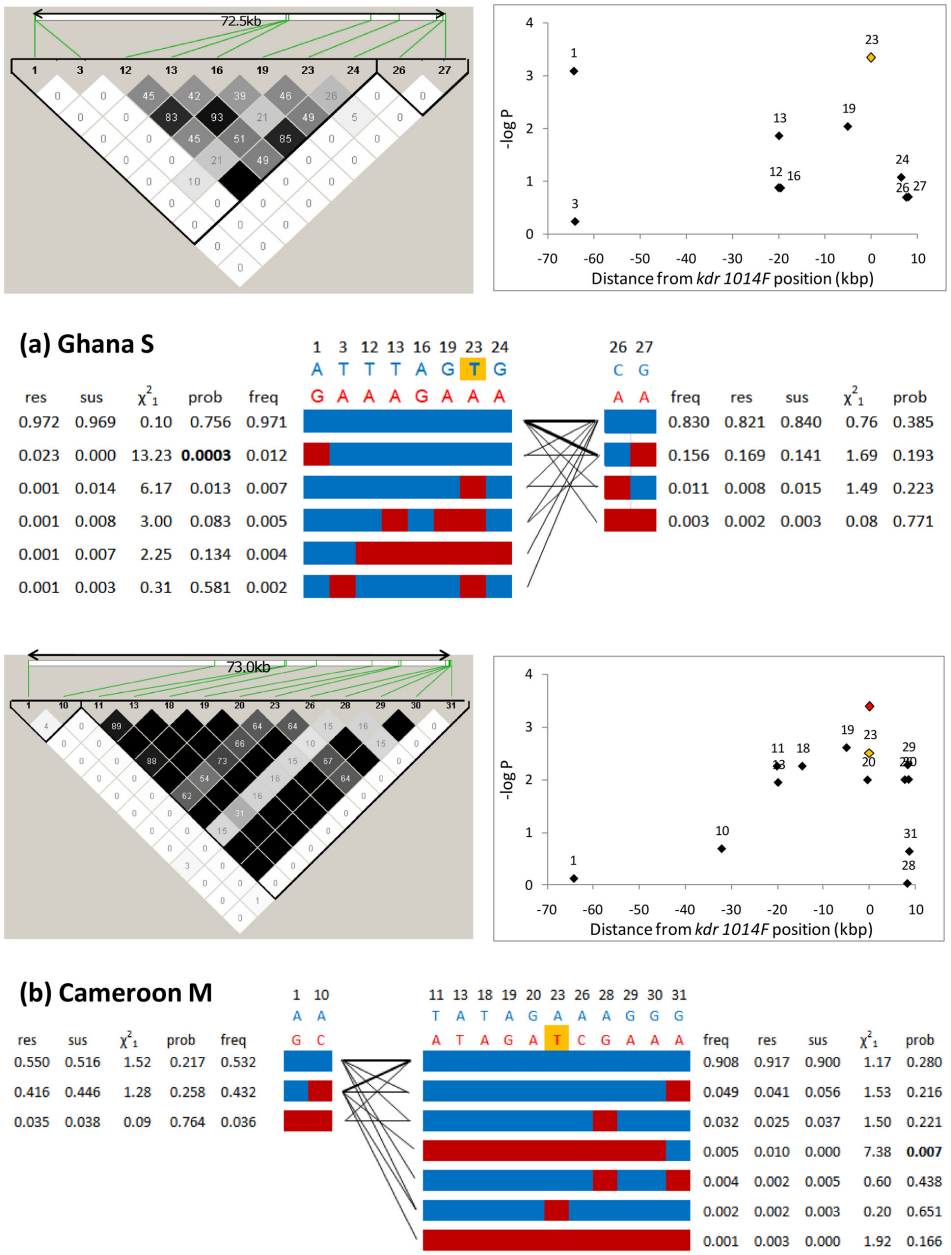
Sodium channel association analyses. Upper left panels in (a) and (b) show pairwise linkage disequilibrium (values are r^2^); green lines show SNP positions on linear scale). Block divisions are created simply to reduce the number of haplotypes by separating highly polymorphic peripheral SNPs from the rest of the (low MAF) SNPs. Upper right panels in (a) and (b) show single SNP association analysis for the sodium channel. Lower panel shows resultant haplotypes for each block with their interconnections (thick lines indicate connection frequency>10%). Total frequencies (freq), frequencies in resistant (res) and susceptible (sus) mosquitoes and haplotype association test statistics are shown. Colours in haplotypes correspond to base colours shown above (*kdr L1014* is highlighted in gold). SNP nomenclature is consistent across plots, with absent numbers monomorphic. In (b) recalculation of single SNP association statistics considering only individuals possessing typical kdr (red block-dominated) haplotypes results in the strongest association of *kdr L1014* (red point in upper left plot).

In the large-sized subsamples - Cameroon M and Ghana S - all SNPs exhibiting high odds ratios (ORs) for permethrin resistance were either very rare or very common (*i.e.* very low minor allele frequency, MAF)(Figure 5). Indeed, although there were 39 and 38 SNPs with ORs>2 in Cameroon M and Ghana S, respectively, none exceeded the frequently-applied cut-off threshold of MAF >0.05 (Table S3). A consequence of this relationship between effect size and polymorphism was that power was generally insufficient to detect significant associations in a single population following correction for multiple testing (Figure 6). Nevertheless, when probabilities were combined across subsamples, three SNPs, all in the 2L voltage-gated sodium channel, were significantly permethrin-associated following strict Bonferroni correction. Although the low combined probability of one rare SNP was underpinned solely by the low probability in the Cameroon S subsample (Table 2), the other two - a known knockdown resistance allele *kdr L1014F*, and another SNP ≈5 kb away (and in strong LD with *kdr L1014F*; Figure 7) – showed useful replication criteria of the same associated allele and comparable odds ratios and P-values in each individual population (Table 2). Whilst several other SNPs exhibited P<0.001, these were unreplicated across samples, which might reflect either population-specific importance or variable efficacy across populations in LD-based detection of a signal from an untyped causal variant. The latter explanation might be particularly pertinent for the two SNPs in unannotated genes toward the centromere of chromosome 2L in Ghana, which are situated in an area of very low SNP-coverage on our array.

**Table 2:** SNPs associated with permethrin resistance at a nominal level of P<0.001.

Chr	Position Ensembl v48	dbSNP rs number	SNPlocation	Gene	AGAP number	Minor allele frequency			Associated allele			Association probability (allelic χ^2^ test)				Odds ratio		
						Cam M	Cam S	Gha S	Cam M	Cam S	Gha S	Cam M	CamS	GhaS	Combined	Cam M	Cam S	Gha S
2R	50992680	5252515	intronic	GSTD7	AGAP004163	0.110	0.205	0.057	**C**	A	A	0.0002	0.73	0.39	0.0033	1.99	1.32	1.24
2R	59757178		non-syn(N->T)	novel^a^	AGAP004659	0.300	0.452	0.455	A	G	G	0.31	0.72	0.0004	0.0047	1.13	1.24	1.52
2R	59842039		5′ UTR	novel^b^	AGAP004660	0.339	0.371	0.278	A	G	G	0.75	0.36	0.0002	0.0031	1.04	1.88	1.64
2L	2358328		intronic	Na channel	AGAP004707	0.036	0.000	0.012	A	-	G	0.74	n/a	0.0008	0.0051	1.11	n/a	17.46
2L	2402689		intronic	Na channel	AGAP004707	0.000	0.018	0.004	-	A	A	n/a	**6**×**10** ^−**6**^	0.13	**1**×**10** ^−**5**^	n/a	n/a	5.34
2L	2417678		intronicor syn^c^	Na channel	AGAP004707	0.006	0.090	0.010	G	G	G	0.003	0.002	0.009	**7**×**10** ^−**6**^	n/a	7.36	6.99
2L	2422652		NS (L-F)^d^	Na channel	AGAP004707	0.010	0.129	0.018	T	T	T	0.003^e^	0.002	0.0005	**5**×**10** ^−**7**^	5.84	6.56	6.58
3R	29226245	5402540	intergenic	CYP4C35/CYP4C36	AGAP009240/AGAP009241	0.315	0.123	0.121	G	G	A	0.15	0.0002	0.43	0.0010	1.19	10.20	1.15

a putative homeotic antennapedia protein (*Aedes aegyptii*, 79% ID) - not pre-identified candidate.

b putative homeotic antennapedia protein (*Aedes aegyptii*, 92% ID) - not pre-identified candidate.

c alternative transcripts: synonymous in two, intronic in one.

d *Kdr L1014F*.

e probable mix of two independent homoplasic associated alleles (see text). P values shown in bold are significant following Bonferroni correction for multiple testing.

### Sodium channel haplotype analysis

Analysis of haplotypes within the sodium channel (target site) gene revealed a more complex picture in both Ghana S and Cameroon M forms than suggested by single SNP analysis alone (Figure 7). In both collections there was a peak of association at or near the *kdr L1014F* SNP position, but in Ghana a second strongly associated SNP was present approximately 65 kb upstream in the first intron of the sodium channel gene. This variant was only present on the same haplotype as the *kdr* allele and its presence alone was associated with a much more strongly resistant haplotype, when compared to the common *kdr*-containing haplotype (Figure 7a). Since the intron 1 variant only occurred in combination with the *kdr L1014F* mutation in Ghana it is unclear whether this might be an additive or epistatic effect. However, the associated intron 1 SNP allele conferred no resistance within a wild type haplotype background in Cameroon M forms (Figure 7b), which argues against a universal additive effect. In Cameroon there were two very similar *kdr*-containing haplotypes, which were only found in resistant individuals. The more common of these was identical to common haplotypes found in Ghana and Cameroon S forms (Table S4), suggesting a plausible route for introgression from S forms. By contrast, a third haplotype featured the *kdr* allele on an otherwise wild type background, consistent with a recurrent mutation at the locus or, less probable, a minimum of two recombination events flanking the 1014 position. Considering only Cameroon M form individuals possessing the ‘typical’ *kdr* haplotypes (i.e. those likely to have a common origin) elevated *kdr L1014F* to the peak of the association signals in the sodium channel, as seen in the Ghanaian S forms (Figure 7). Thus, in both the Ghana and Cameroon samples there was evidence that the *kdr* mutation does not act alone but is at least partially dependent upon local haplotypic background. We detected no evidence that any portions of the sodium channel which we genotyped might be duplicated since all SNPs were in Hardy-Weinberg equilibrium (Table S3). Finally, sequencing confirmed that the second known *A. gambiae* knockdown resistance mutation, *kdr L1014S* (not typed on the array), was present in the Cameroon M and S samples. We discontinued plans to sequence sufficient individuals to permit genotype imputation because the allele previously linked with resistance [7] showed no resistance-association whatsoever (S forms, OR = 0.4, N = 29; M forms, OR = 0.1, N = 16).

## Discussion

This is the first large-scale association study of insecticide resistance in wild *Anopheles* populations and provides important novel insights into target site resistance as well as the sources and consequences of population stratification and genomewide LD level. Even at a fine scale average LD was very low with consequent impacts on power to detect untyped causal variants via LD with typed markers because of the direct inverse proportionality between r^2^ and sample size in association analyses. Comparable data on LD from other wild insect populations are quite limited. Using a 1536-SNP array Whitfield et al. [34] reported average LD of r^2^>0.4 at a scale of ≤5 kb in European honeybees *Apis m. mellifera*, which have a recent history of severe population bottlenecks, but r^2^≈0.1 for wild African honeybees *A. m. scutellata.* The latter is entirely comparable to our estimates for wild *A. gambiae*. Though direct quantitative comparisons are difficult since r^2^ is rarely used, typical LD in *Drosophila* also appears to be very low [35]. Thus the challenges in applying LD mapping techniques to wild populations of *A. gambiae* are likely to apply to other insects and probably to wild populations of many taxa.

The strongly inverse relationship between effect size and MAF we observed is in accord with recent model predictions advanced as an explanation for why the plethora of GWA studies has rarely detected major effect variants underlying common human diseases [36]. In contrast, we did detect with statistical confidence (P≈10^−6^) association of major effect variants in the insecticide target site gene, though this required combined analysis of the three subsamples. Indeed, given the low MAF and loss of power with genomic control, a sample size increase of approximately 30% (assuming an identical odds ratio to that observed) would be required to obtain a P-value less than the Bonferroni–corrected α level for the *kdr L1014F* in the Ghanaian sample alone, where permethrin resistance was most pronounced. Reduced power resulting from low MAFs was ameliorated to some extent by direct typing of a causal SNP, although the *kdr L1014F* association was also detected indirectly via LD with a SNP 5 kb away. This situation may be exceptional, however, because the high LD within the 2L voltage-gated sodium channel likely results from a combination of strong recent directional selection acting upon *kdr L1014F* and low recombination rate in an area relatively close to the 2L centromere [37]. The low MAF but relatively high LD illustrates a yin and yang of LD mapping of strongly-selected phenotypes: selective sweeps reduce local MAFs (and power) and increase LD (and power).

Major effect target site variants, especially *kdr L1014F*, are clearly of major importance in permethrin resistance, and it is noteworthy that in populations differing dramatically in both resistance levels and *kdr L1014F* frequencies this was the only consistently associated SNP. However, and despite the large effect sizes observed, it is premature to conclude that target site variants play the pre-eminent role in permethrin resistance. Power to detect anonymous causal variants depends heavily on locally high LD, which, with the notable exception of the 2La inversion region [38] was absent from most of the *A. gambiae* genome. In Cameroon M and Ghanaian S forms, three potentially promising candidate SNPs were detected (at P<0.001) toward the centromere of chromosome 2R, though each exhibited a low-moderate odds ratio (OR<2). It is entirely plausible that weak signals of association (moderate P-values and/or low-moderate ORs) in regions of low LD or low marker coverage could result from weak detection of causal variants, which actually exert a strong effect on the phenotype. Alternatively it is possible that signals of association toward the centromere of chromosome 2R could emanate from the peri-centromeric region of chromosome 2L and perhaps the sodium channel gene therein, with restricted recombination generating extended LD across centromere [39]. Follow-up work to examine these hypothesis using both a recently-developed high density SNP array and whole genome sequencing are currently underway. Until such data are available, the possibility will exist that metabolic variants of major effect, suitable for predictive diagnostic screening in the same way as target site variants, are masked by low LD and remain undetected.

Widespread high frequency of *kdr L1014F* in West African S forms, coupled with evidence for introgression of *kdr L1014F* from the S to M molecular form presented here and by other authors [37], [40], [41], [42], and dramatic recent increases in *kdr L1014F* frequency in M forms in West Africa [37], [43] presents a disturbing scenario for pyrethroid-based vector control programmes. Evidence for direct impacts of *kdr*-mediated insecticide resistance on malaria transmission are limited, and somewhat equivocal at present [44], [45] but large-scale trials required to provide such data are now underway. Although a plethora of studies have examined the association of *kdr* (*L1014F* or *L1014S*) with pyrethroid or DDT resistance in field populations of *A*. *gambiae* [9], almost all have typed the known *kdr* mutation alone as resistance candidates. Our study is the first to simultaneously evaluate association of *kdr*, multiple other sodium channel SNPs, and SNPs in many other plausible candidate genes. In so doing, our study has been able to demonstrate, for the first time to our knowledge in *A. gambiae*, the importance of haplotypic background on *kdr L1014F* resistance association. In Cameroon M forms two *kdr* haplotypes arose via either recurrent mutation (see also [42], [46]) onto, or perhaps recombination with, a wild type background, with some evidence for alteration of resistance levels, albeit limited by low haplotype frequencies. In Ghana S forms an additional SNP within the sodium channel raised resistance levels above that of *kdr L1014F* homozygotes. The effect of this resistance-associated intron 1 SNP effect resembles that of the *M918T super-kdr* mutation, which co-occurs with *kdr L1014F* in other insects [47]. However, given its distance from the 918 amino acid codon and the genotyping of multiple closer SNPs, *M918T* is almost certainly not the causal SNP with which the intron 1 SNP is in LD. Replication of this association signal is now important and we are currently working to identify the functional variant for screening in additional populations. Nonetheless, the haplotype-dependency of *kdr* argues for the incorporation of additional sodium channel SNPs into routine insecticide resistance monitoring programmes.

The need for simultaneous assessment of molecular form and insecticide resistance was very clear with strong covariation between molecular form and permethrin resistance in our samples. This is not a standard practice at present in resistance test reporting but needs to become so. Indeed M *vs.* S form represented the major axis of stratification, with very high differentiation throughout the genome. Our data do not actually contradict results from single feature polymorphism (SFP) analyses, which found significant differences only at a few genomic regions, particularly regions near the centromeres of chromosomes X and 2L [24], [25] because these were tailored toward detection of large regions of extreme divergence (which we also detected). Our results demonstrate that molecular forms are differentiated at a high and consistent enough level throughout the genome to cause massive inflation of test results, which could not be corrected by statistical genomic control. As long as the need to partition molecular forms in any kind of DNA- or RNA-based association study of *A. gambiae* is recognised, many false positives can be readily avoided by the use of simple PCR-based diagnostics *a priori*. Similarly, a simple PCR diagnostic [31] is available for the second major source of structure within samples, 2La inversion polymorphism. Whilst this had no impact on our results because permethrin resistance did not co-segregate with 2La inversion karyotype, the extreme differentiation between karyotypes in parts of this region (as reported previously [38]) could be problematic for other populations or phenotypes, and some method of stratified or corrected association testing may be required [48]. The source of stratification in the Ghana collection was cryptic and not captured by cluster analysis, however, it is encouraging that genomic control removed this problem and permitted use of all data from S form samples.

### Conclusions

Our data illustrate that association mapping is difficult but feasible for wild *A. gambiae* populations, at least for major effect variants and especially, where LD is enhanced by selective sweeps. Therefore, whilst elucidation of the full genetic architecture of insecticide resistance and other medically important traits will likely have to wait until association mapping by whole genome sequencing becomes economically feasible for *A. gambiae*, major effect variants that will be of greatest utility for predictive diagnostic screening can be identified. Although there was some degree of portability of LD across subsamples, low LD effectively necessitates, as well as facilitates, the identification of functional variants prior to testing for replication in multiple populations. This contrasts with the European human GWAS methodological template, as does the lack of feasibility of tagging SNP-based arrays and utility of a HAPMAP-style reference database for *A. gambiae* and other species with comparable LD characteristics. This inappropriateness of the European human GWAS model for *A. gambiae* mirrors that encountered in GWA studies of malaria susceptibility in low-LD African human populations [19], [20]. It is both encouraging, and perhaps fitting, that future association studies of medically important traits in African mosquitoes are likely to benefit from methodological developments arising from studies of their African human hosts.

## Materials and Methods

### Sample collection and insecticide resistance testing

In Cameroon, samples were collected in July-August 2006 from within an area of approximately 1 km^2^ in Yaoundé (03° 52′ 0′′N 11° 31′′ 0′′ E). Ghanaian samples were collected in October-November 2006 from an area of approximately 28 km^2^ around Dodowa, Greater Accra (05° 53′ 00′′N 00°00′ 00′′W). Larvae were collected from breeding sites using ladles following a protocol designed to reduce relatedness among samples, whereby numbers collected were scaled to the size of the habitat, and, wherever possible, collections from the same habitat were separated by 3–4 days. Larvae were raised in field insectaries under ambient conditions and fed flake fish food twice daily until pupation, at which point they were transferred to emergence cages. Each cage housed a daily batch of pupae and was provisioned with cotton wool soaked in 10% sugar-water. Following eclosion, males were removed. Permethrin resistance phenotypes were determined at 3–5 days post-eclosion.

Classification of permethrin resistance phenotypes is described in detail elsewhere [11] but briefly involved: (1) prior computation of population-specific adult female LT_50_ curves for permethrin using standard WHO 0.75% permethrin papers and testing tubes, with samples collected in the same way and from the same area; (2) exposure of 3–5 day old adult females to permethrin for the population-specific LT_50_ time, with transfer of all mosquitoes to insecticide-free holding tubes post-exposure; (3) classification of females as resistant (alive) or susceptible (dead) 24 h post-exposure. All phenotyped females were stored individually in pierced 0.2 ml Eppendorf tubes over silica gel.

### Design of Illumina assay

Our 1536 SNP array (Table S1) was designed primarily to cover 266 candidate genes potentially related to insecticide resistance. Most of these genes are represented on the *A. gambiae* Detox microarray chip [49], thus providing complimentary genotyping and expression arrays. 1196 SNPs were located within or very near to candidate genes (mean coverage 4.5 SNPs/candidate) with the remaining 340 SNPs, located in intergenic regions or non-candidate genes, serving as controls.

### Sample preparation and screening

DNA from the individual dried mosquitoes was extracted using the Qiagen DNEasy kit and quantified using the PicoGreen fluorimetric assay (Invitrogen). Since DNA extracts from individual mosquitoes typically contain insufficient DNA for high throughput genotyping assays [50], whole genome amplification was required using 50 ng of DNA extract as template for the GenomiPhi V2 DNA Amplification Kit (GE Life Sciences). Following whole genome amplification, each sample was quantified using PicoGreen and diluted to yield 50 ng/µl: 5 µl served as template for the Illumina GoldenGate assay, which we ran on an Illumina Beadstation GX according to the manufacturer's protocols. To provide a test of both repeatability and effects of whole genome amplification we genotyped 11 samples using both amplified and unamplified DNA (on separate arrays). Repeatability was appropriately high, with a mean error rate of 0.3%.

Morphological checking of all samples during field collections had confirmed their identity as *Anopheles gambiae s.l.* A PCR-RFLP molecular diagnostic of X chromosome rDNA variation [29] was used to determine species identity within the *A. gambiae s.l*. group and *A. gambiae s.s.* molecular form (M or S), prior to GoldenGate genotyping: only *A. gambiae s.s*. were included. A portion of samples, most of which were chosen because of apparent inconsistencies between Illumina data and molecular form were also screened using an alternative assay for molecular form identification which types variation at a Sine marker on the X chromosome [30] and screened a second time using the rDNA PCR-RFLP diagnostic.

### Data analysis

Genotyping arrays were scored using Beadstudio v3.2 (Illumina Inc.): all automatic calls were checked manually and occasionally amended to increase separation among cluster boundaries (resulting in greater call certainity more more missing values), though in most cases such SNPs were excluded. Of the 1536 SNPs on the array 886 were could be scored reliably in each sample collection and were polymorphic in at least one (Table S1): only these SNPs were used in the present analysis. Owing to massive stratification (see Results), deviation from Hardy-Weinberg proportions alone was not used as an exclusion criterion prior to cluster analysis. Individual Bayesian cluster analysis of genotypes at the control (non-candidate) SNP loci was performed by BAPS 5.2 [51]. Multiple runs of BAPS were performed to obtain the optimum clustering solution for each sample collection. Confident assignment of an individual to a cluster was determined to have failed if the probability associated with movement of a genotype from its optimum cluster to any other was greater than a Bonferroni-corrected critical alpha level. Relative distances among clusters, based on the Kullback-Leibler distance produced by BAPS 5.2, were visualised using neighbour-joining trees created by Phylip 3.68 [52] and drawn by TreeView 1.6.6 [53] or by multidimensional scaling using SPSS 14. Differentiation at each SNP (candidate and control) was computed as F_ST_ [54] in Genepop 4 [55]. Linkage disequilibrium among SNPs was computed (as r^2^) and visualised using Haploview 4.1 [56]. Haploview was also used for haplotype reconstruction and haplotypic association tests. The genomic control statistic, λ, where theoretical χ^2^_1_ =  λ.χ^2^_1_ was calculated from association tests [57]. Only independent control SNPs were used (nominally determined as pairwise r^2^≤0.1); in cases of non-independence the SNP(s) with lower genotyping success rate(s) was discarded. SNPs yielding Hardy-Weinberg test probabilities lower than the Bonferroni corrected critical α were excluded from both genomic control statistic computation and association tests (following partitioning of molecular forms). Chi-squared tests of single SNP and haplotypic association were computed using Haploview 4.1. Probabilities were combined across populations using Fisher's method [58].

## Supporting Information

Figure S1 Alternative version of cluster analysis for Ghana S forms. Population structure determined by cluster analysis of Ghanaian S form genotypes comprising of control SNPs situated outside of the 2La inversion region.
(0.10 MB DOC)

Figure S2 Linkage disequilibrium tri-plots. Plots of pairwise linkage disequilibrium for all chromosomes in each population.
(2.80 MB DOC)

Table S1 SNPs used on the 1536 Illumina array. Full list and description of SNPs on the 1536 Illumina array.
(0.46 MB XLS)

Table S2 Clustering and 2La karyotypes. Relationship been multilocus genotype clusters and 2La karyotypes in each major subpopulation.
(0.04 MB DOC)

Table S3 Association test results. Full list of permethrin-association test results in each population.
(0.31 MB XLS)

Table S4 Full list of sodium channel haplotypes in each sample collection.
(0.05 MB XLS)
