# Supplementary material for: Association Mapping of Insecticide Resistance in Wild Anopheles gambiae Populations: Major Variants Identified in a Low-Linkage Disequilbrium Genome
Source: PLoS One. 2010 Oct 1;5(10):e13140. doi: 10.1371/journal.pone.0013140 (PMC2956759; doi:10.1371/journal.pone.0013140)
Supplement: Figure S1 — Alternative version of cluster analysis for Ghana S forms. Population structure determined by cluster analysis of Ghanaian S form genotypes comprising of control SNPs situated outside of the 2La inversion region. (0.10 MB DOC) [file pone.0013140.s001.doc]

**Figure S1.** Alternative version of cluster analysis for Ghana S forms. Population structure determined by cluster analysis of Ghanaian S form genotypes comprising of control SNPs situated outside of the 2La inversion region. (a) Neighbour joining tree (based on K-L distance as in Figure 2) of clusters identified with numbers of genotypes shown. Differentiation at all SNPs (N=886) among (b) the three major clusters (G8-10 in (a)), (c) all minor clusters. Note that whilst small sizes of the clusters in (c) will produce high sampling error in F_ST_, there is no localisation of signal to particular genomic regions (cf. (b)).


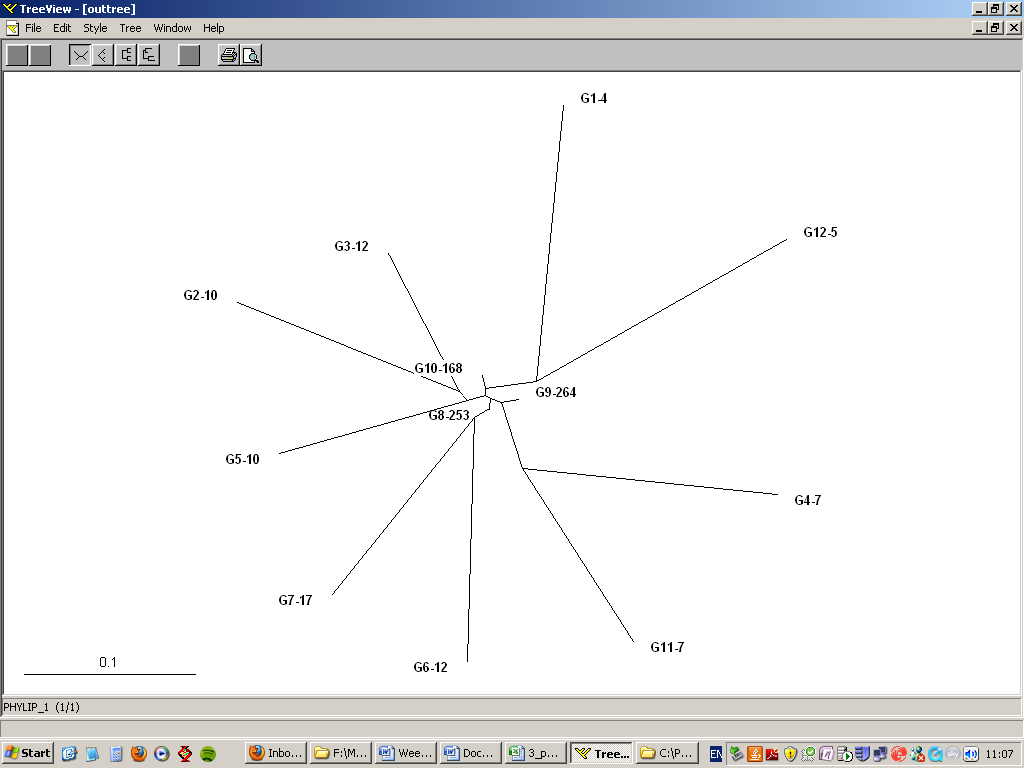


**a**

**b**

3R/3L centromere

≈6.5Mb

2Rb inversion region

**c**
