# Supplementary material for: Association Mapping of Insecticide Resistance in Wild Anopheles gambiae Populations: Major Variants Identified in a Low-Linkage Disequilbrium Genome
Source: PLoS One. 2010 Oct 1;5(10):e13140. doi: 10.1371/journal.pone.0013140 (PMC2956759; doi:10.1371/journal.pone.0013140)
Supplement: Figure S2 — Linkage disequilibrium tri-plots. Plots of pairwise linkage disequilibrium for all chromosomes in each population. (2.80 MB DOC) [file pone.0013140.s002.doc]

**Figure S2. Linkage disequilibrium tri-plots.**

***Key***

Triplots show linkage disequilibrium between pairs of SNPs, based on r^2^ (high r^2^=dark; low=light)

Green lines show SNP positions on a linear physical map of chromosome indicated by scale bars, which show 5 Mb intervals – note the differences in scale and SNP count among plots, especially for the X chromosome.

*T* = telomere position; *C* = centromere position

Sample sizes:

Cameroon M = 673

Cameroon S = 62

Ghana S = 770

**Chromosome 2R (284 SNPs)**

Cameroon M form

*T*

*C*


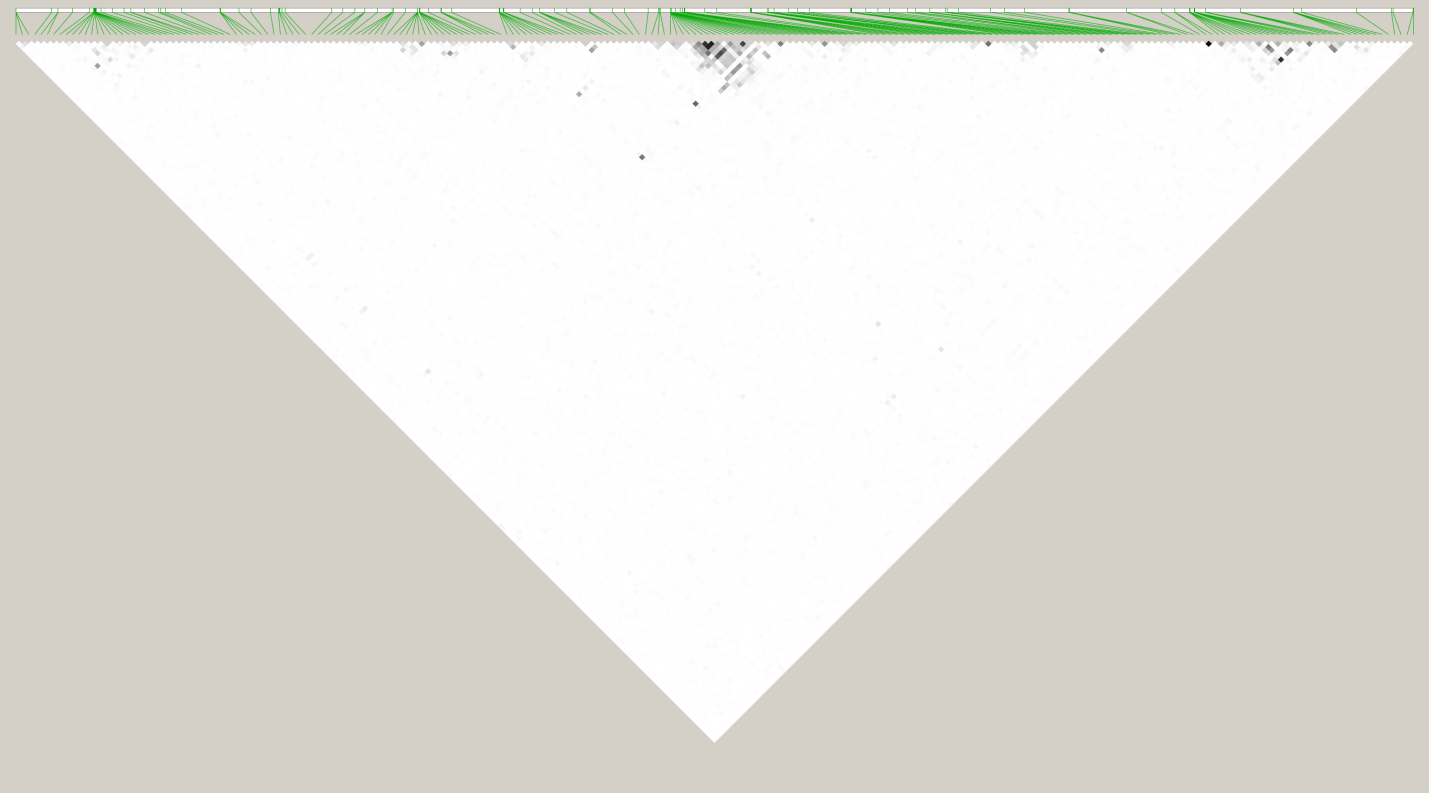


Cameroon S form


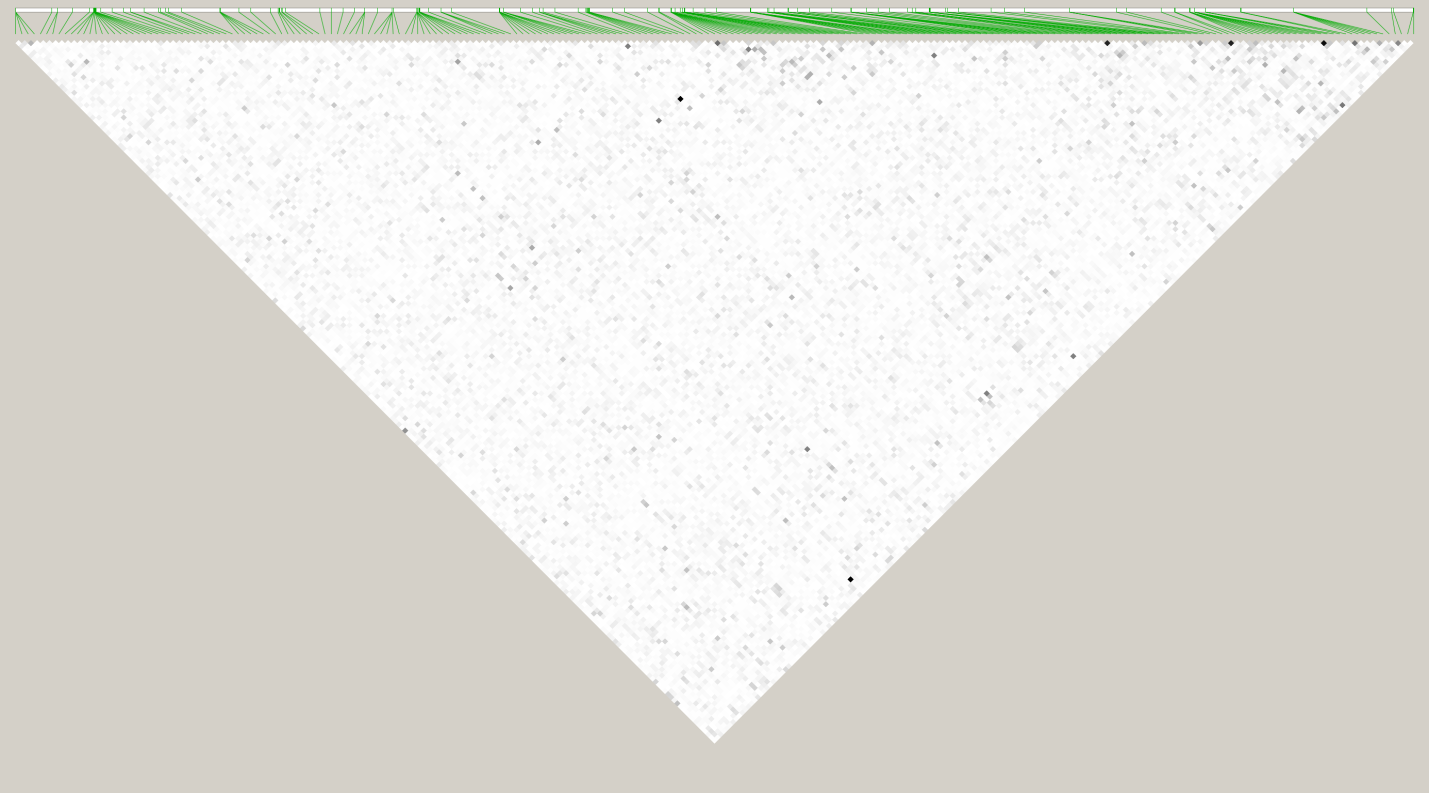


*T*

*C*

Ghana S form


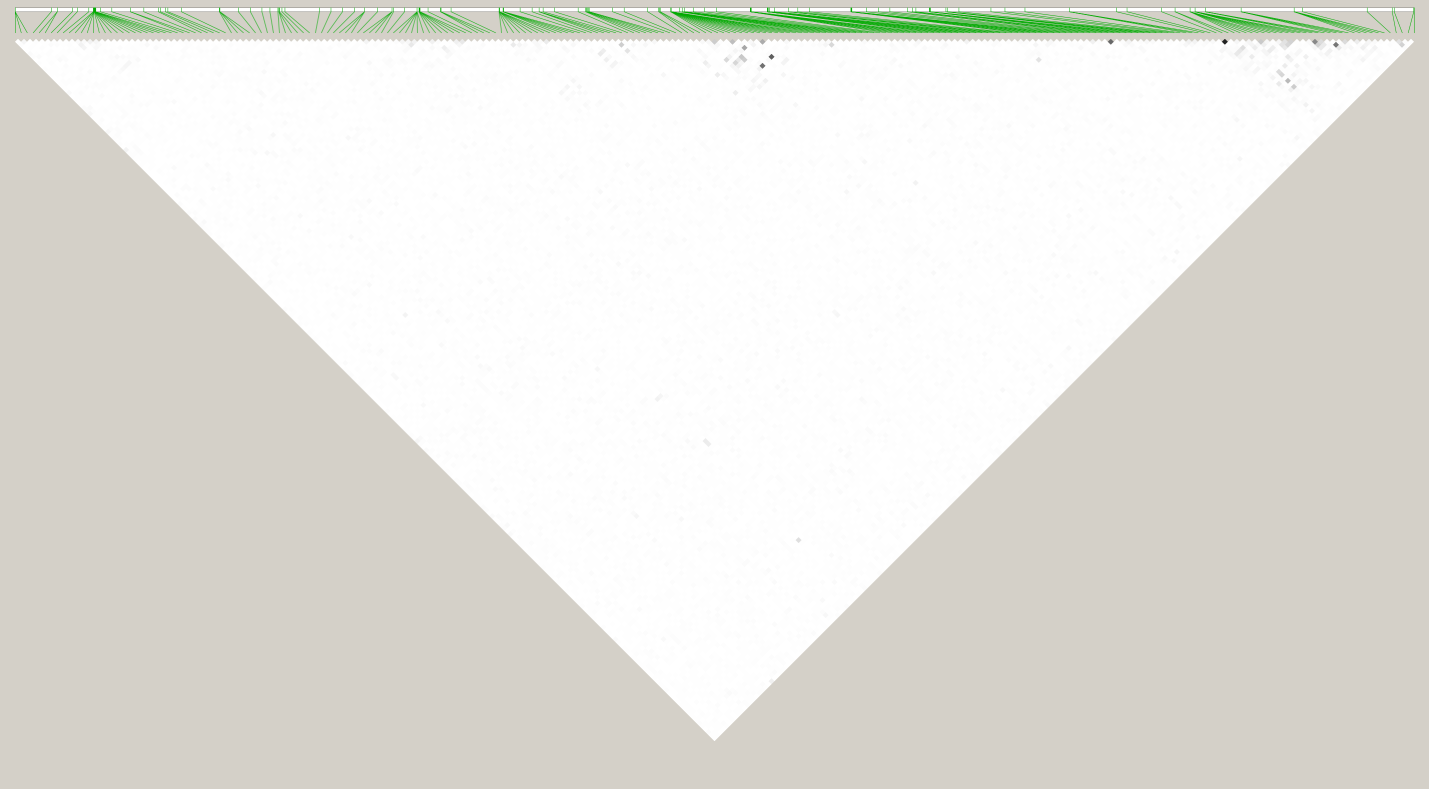


*T*

*C*

**Chromosome 2L (211 SNPs)**

L N

Cameroon M form


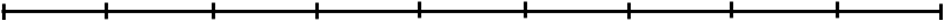


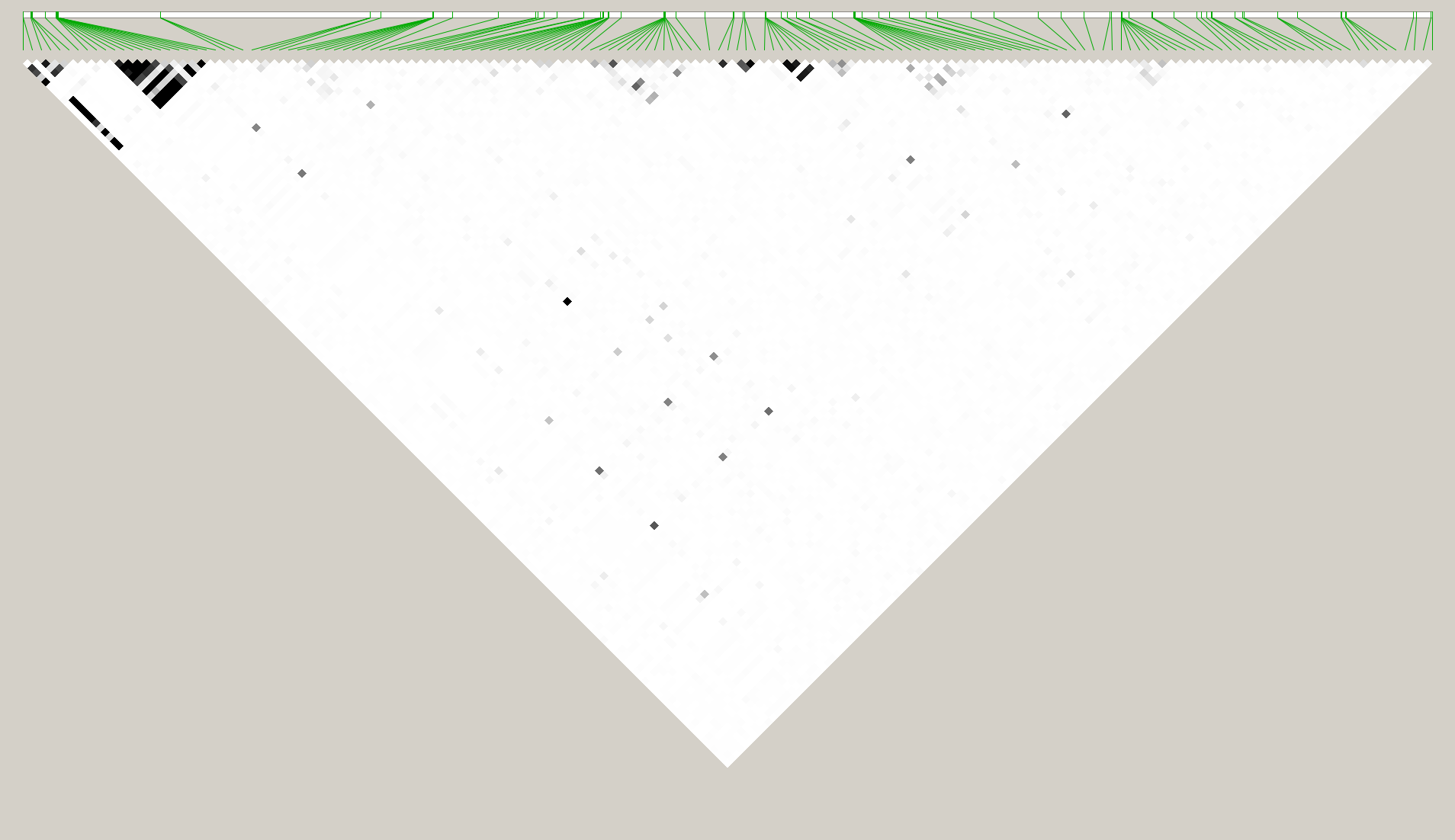


*C*

*T*

N

Cameroon S form


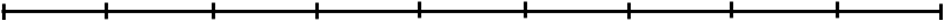


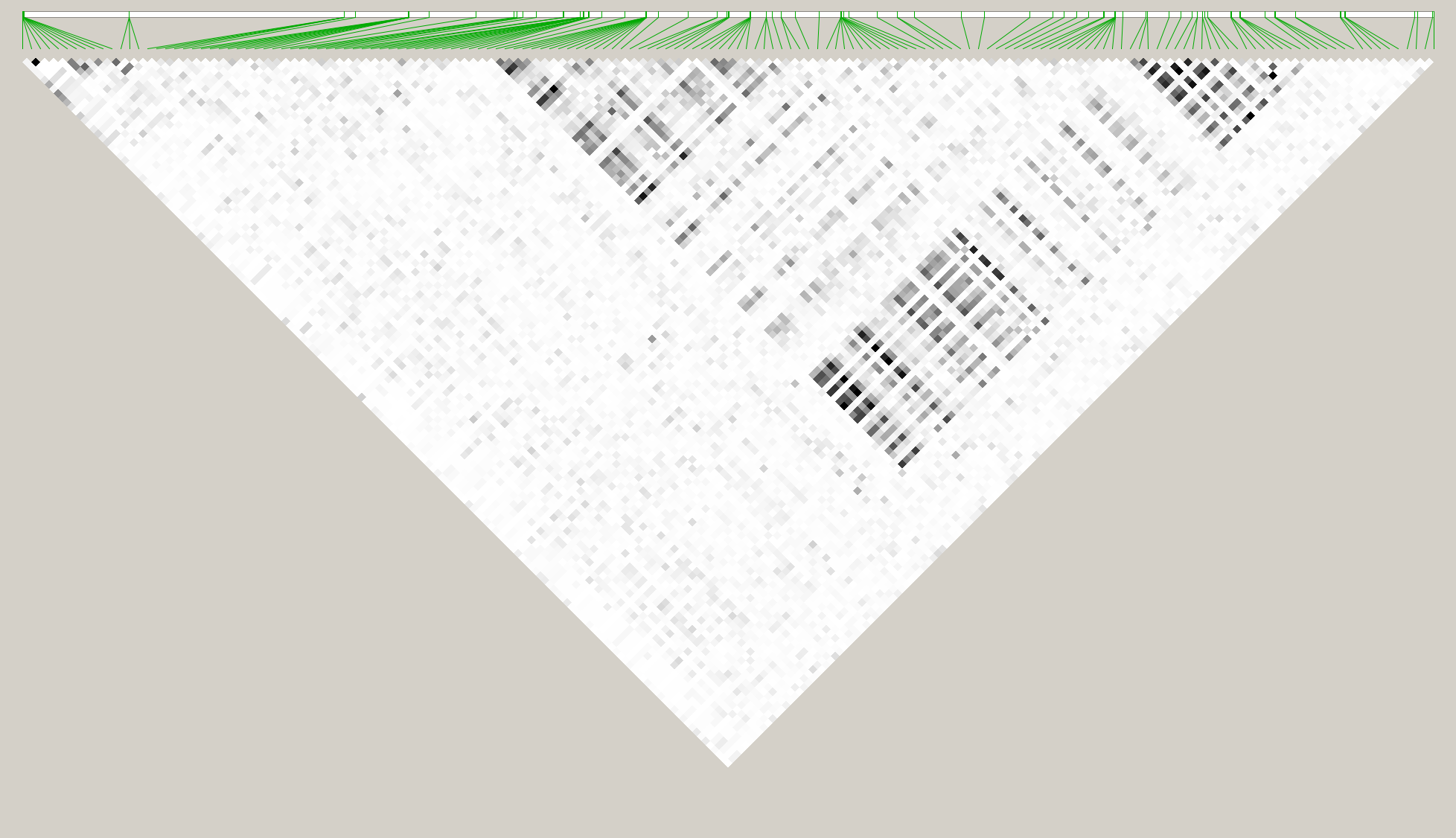


*C*

*T*

Ghana S form

L N


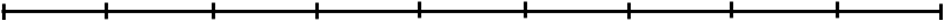


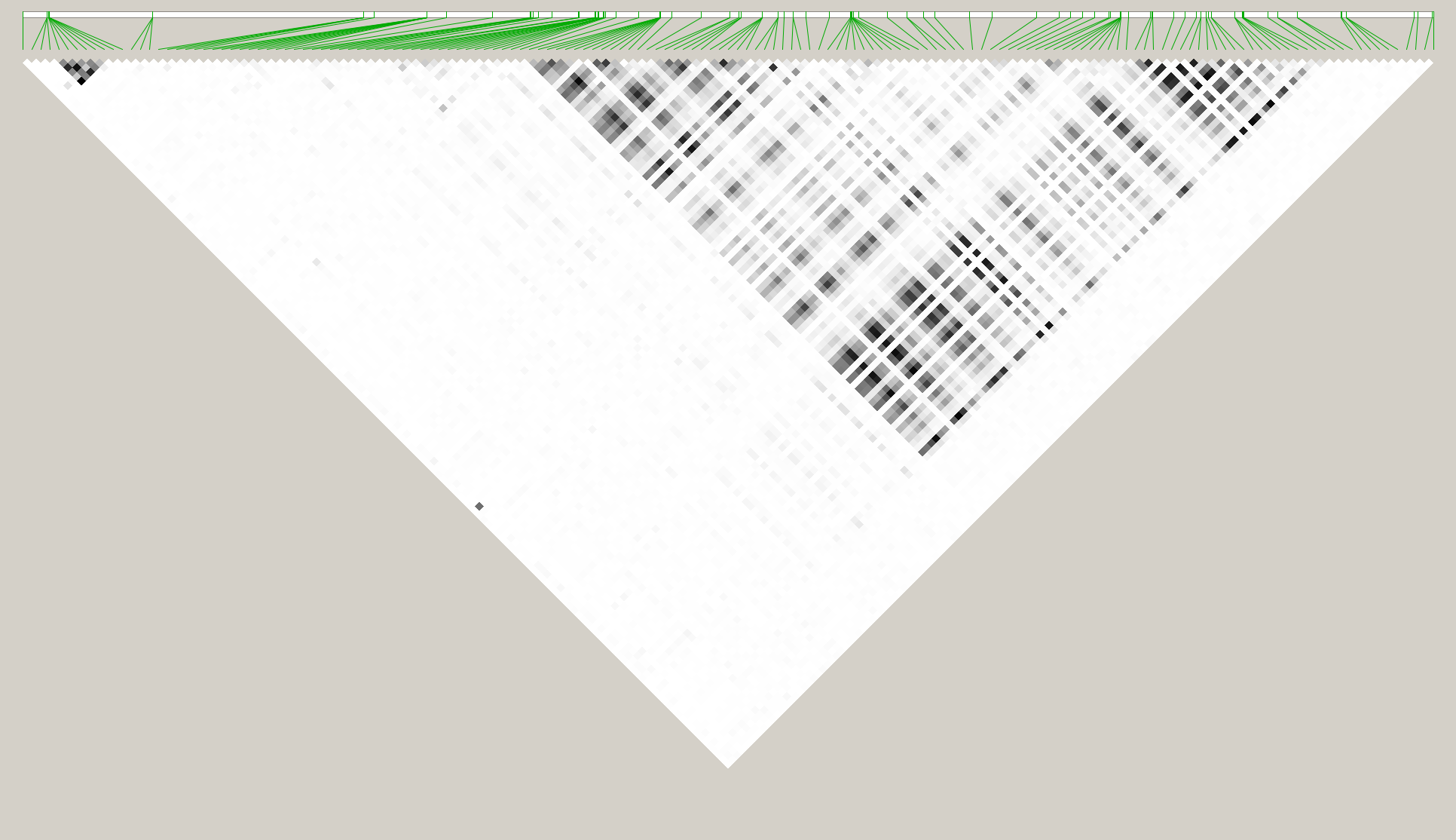


*C*

*T*

***Additional key for chromosome 2L***

L= *Lim* gene (within 2L island of speciation: fixed/ near fixed differences between forms)

N= sodium channel gene (insecticide target site)

Purple bar=2La inversion region

**Chromosome 3R (164 SNPs)**

Cameroon M form


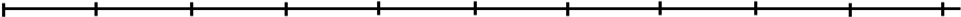


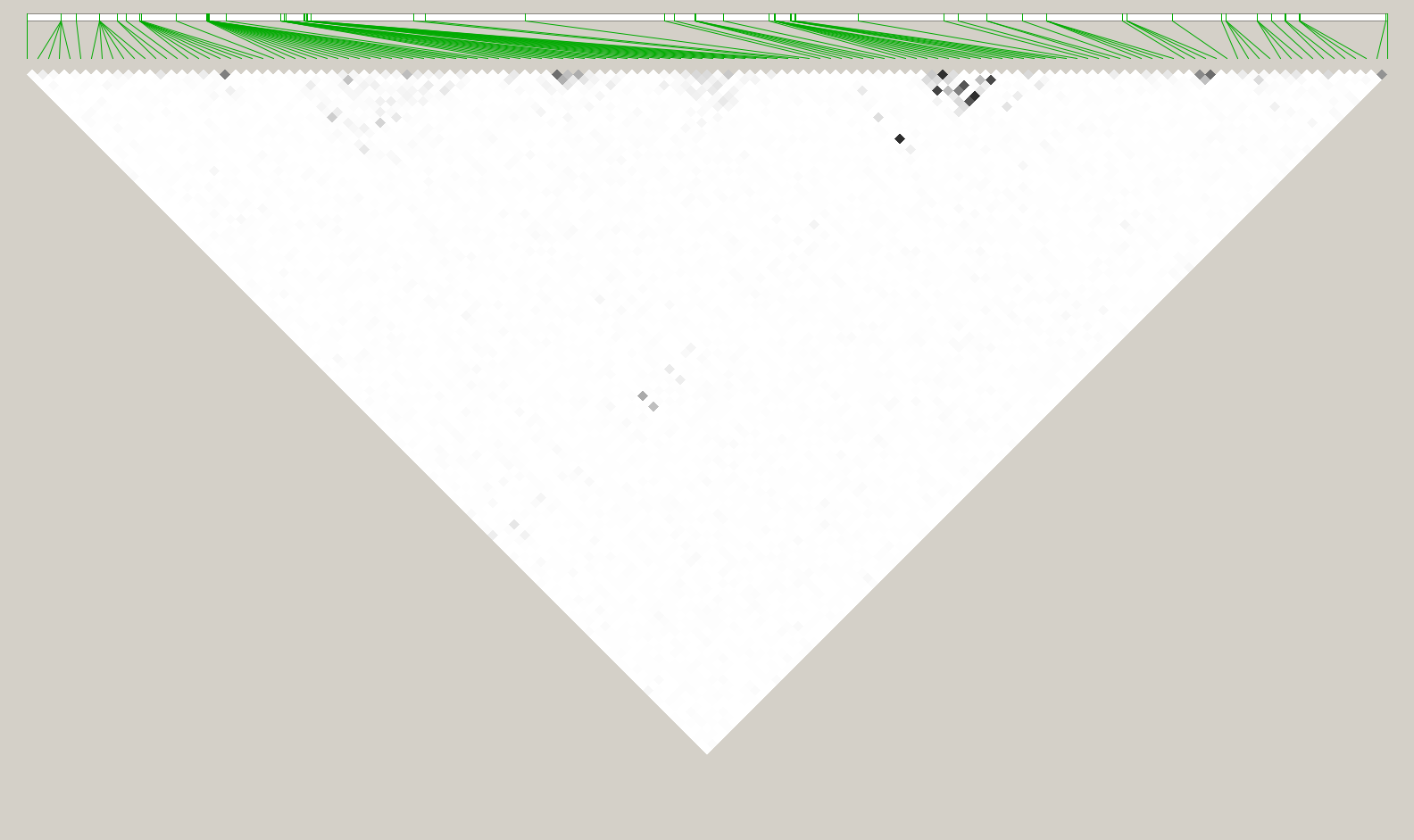


*T*

*C*

Cameroon S form


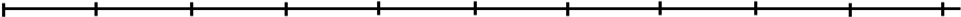


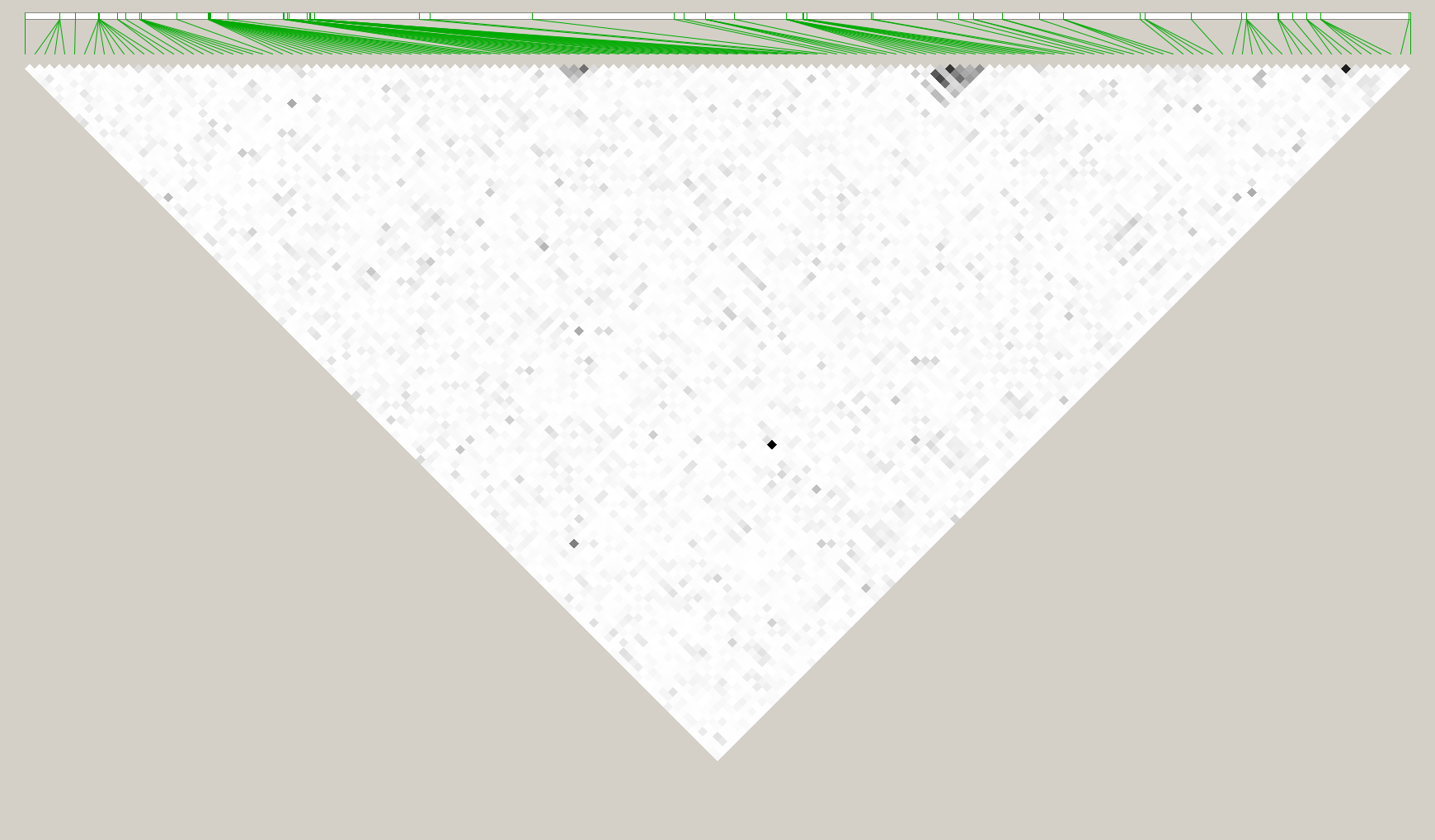


*T*

*C*

Ghana S form


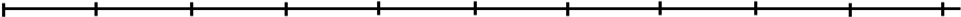


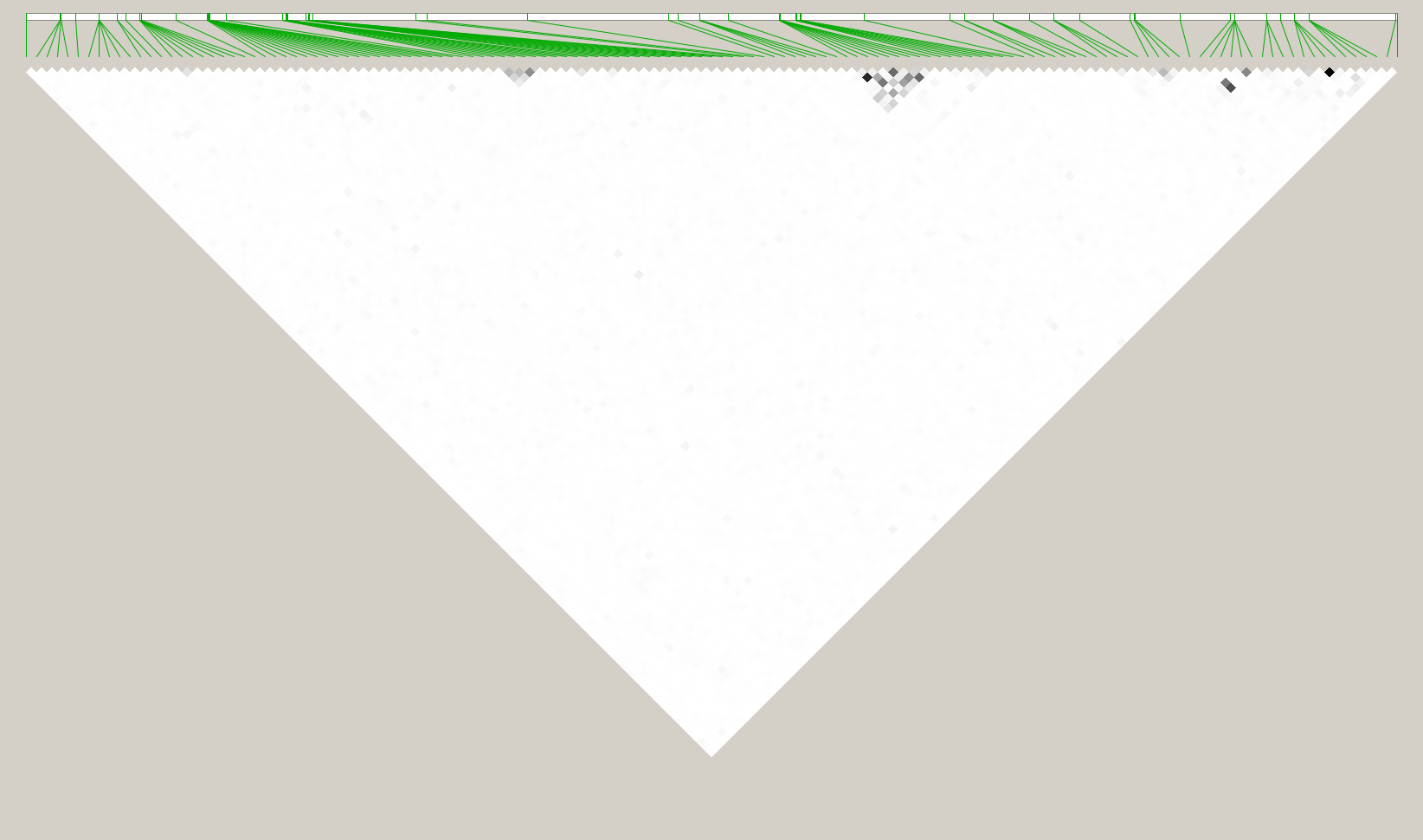


*T*

*C*

**Chromosome 3L (145 SNPs)**

Cameroon M form


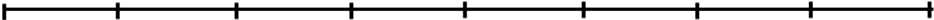


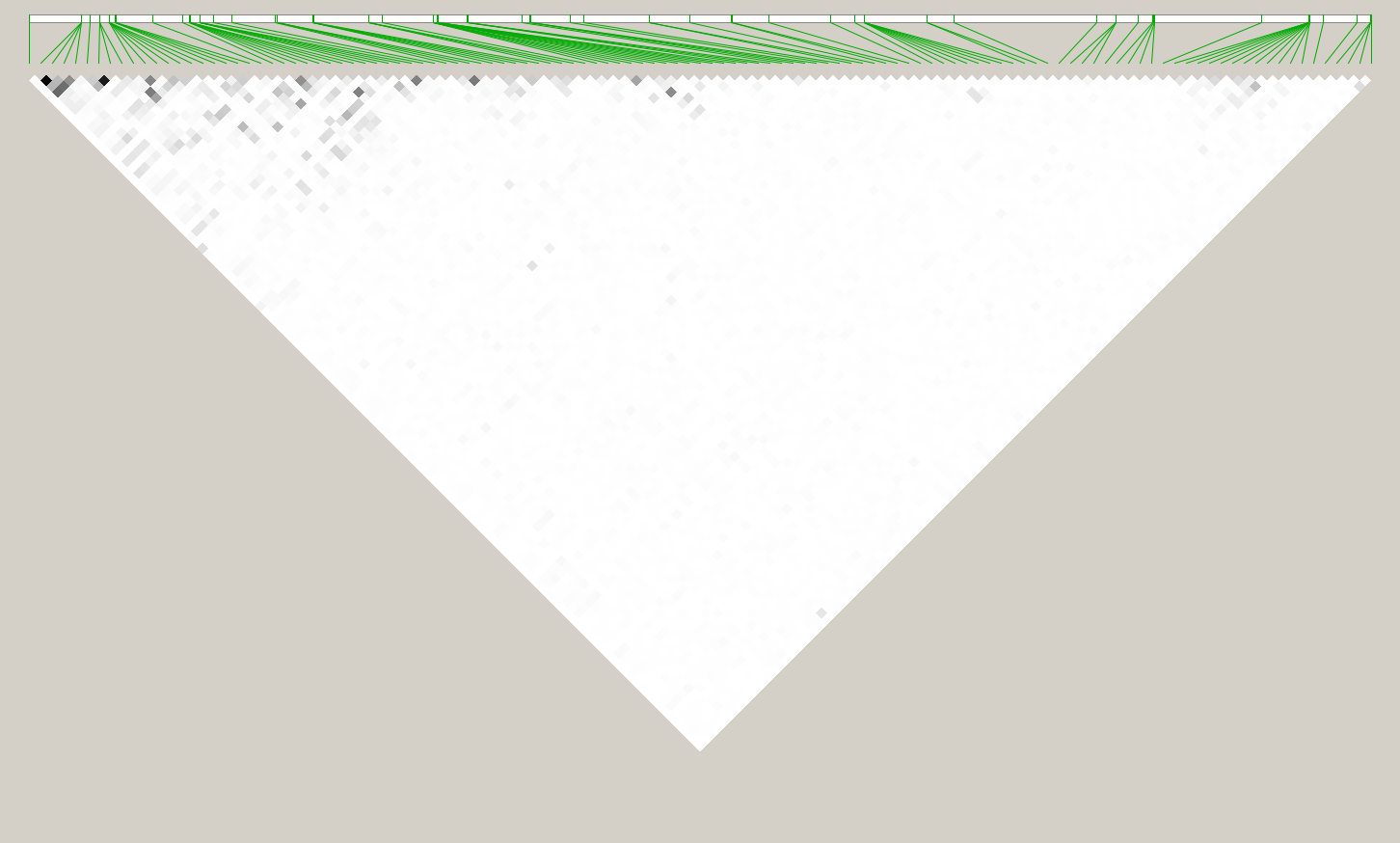


*C*

*T*

Cameroon S form


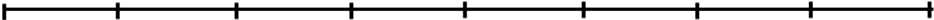


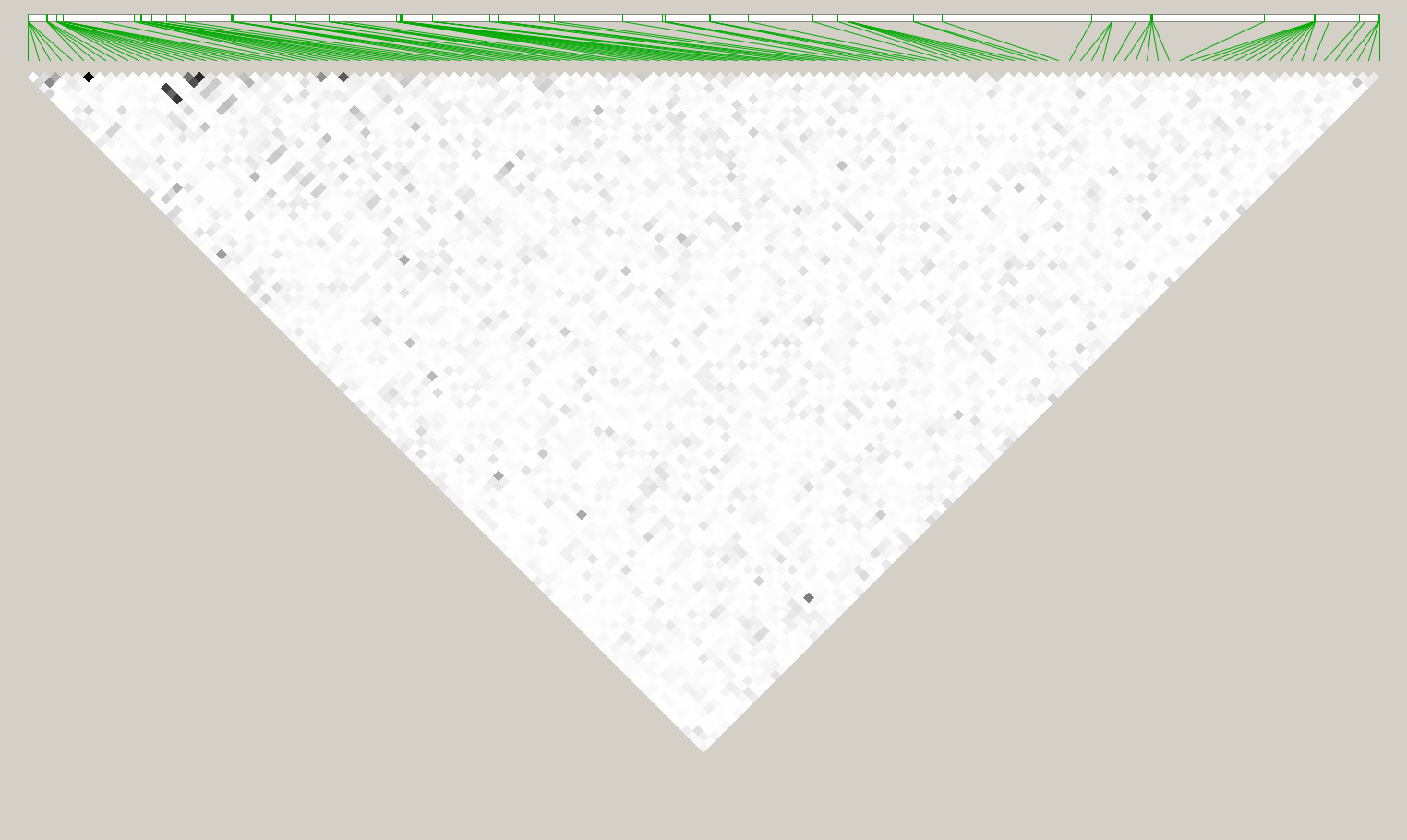


*C*

*T*

Ghana S form


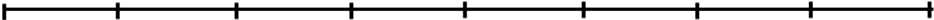


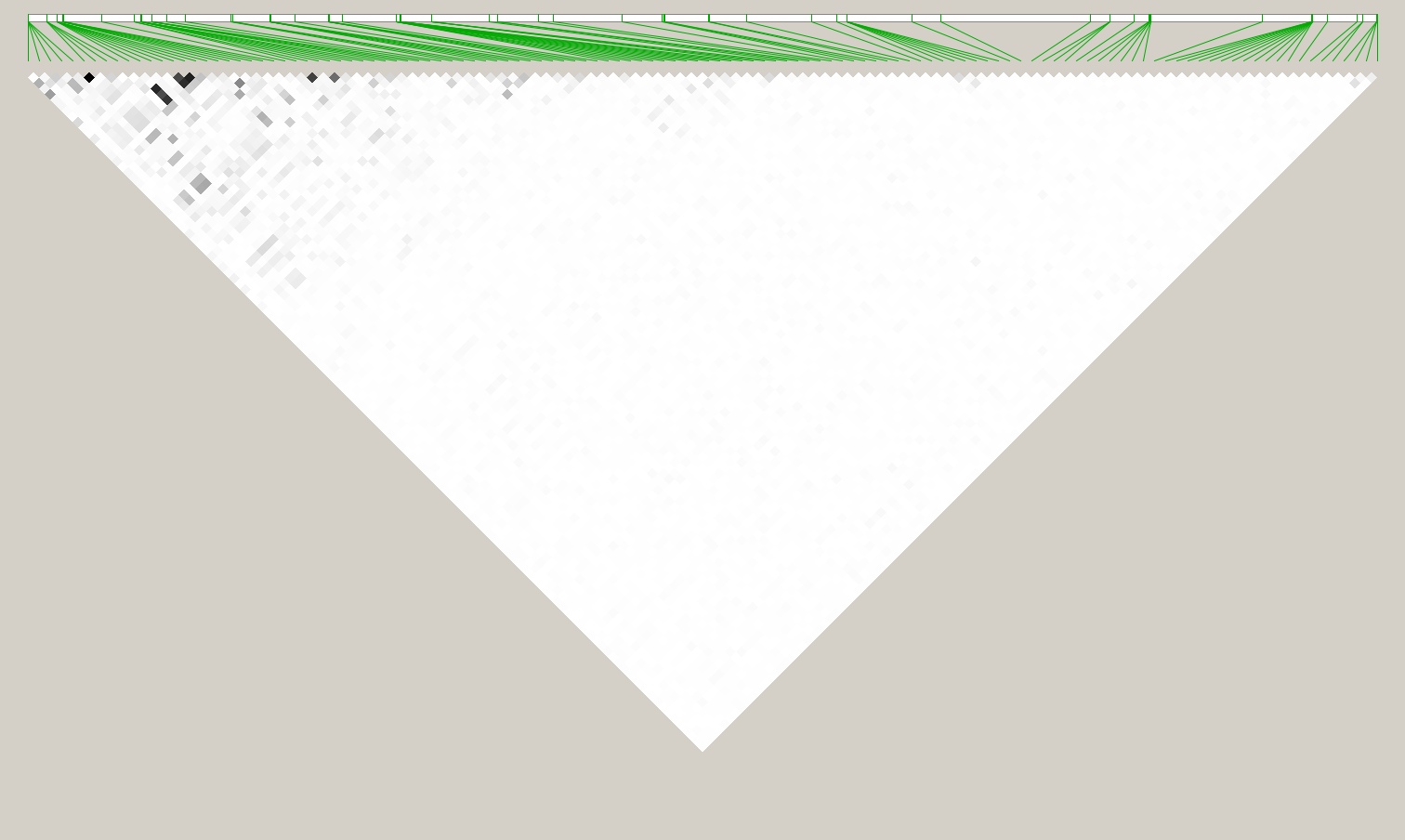


*C*

*T*

**Chromosome X (82 SNPs)**

Cameroon M form


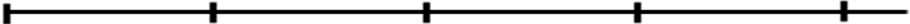


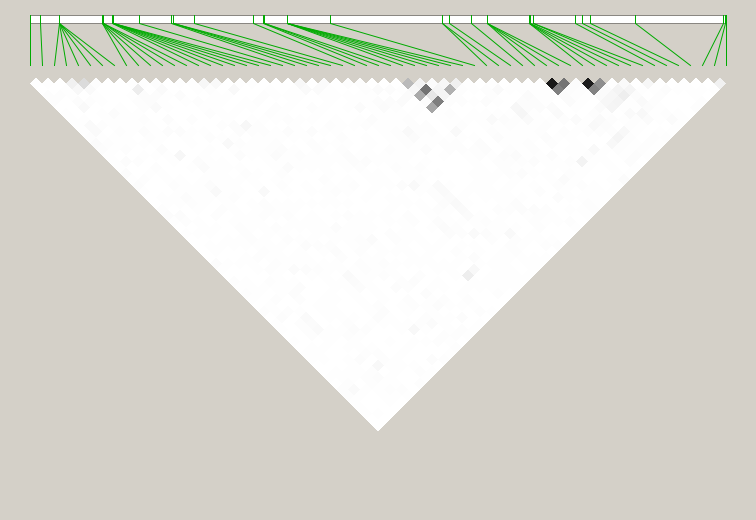


*T*

*C*

Cameroon S form


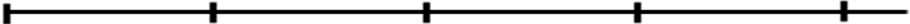


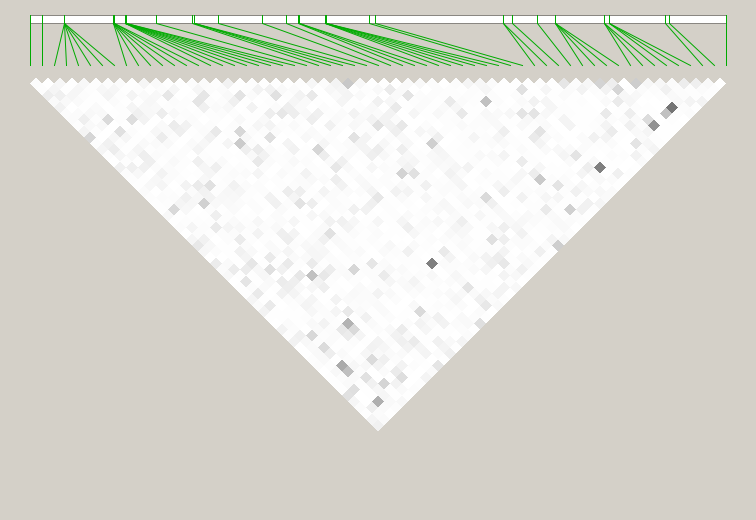


*T*

*C*

Ghana S form


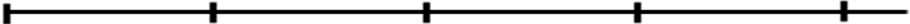


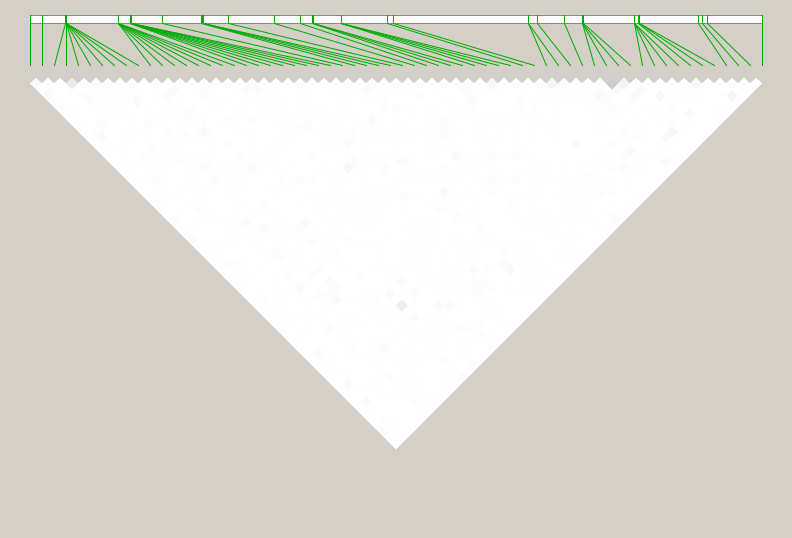


*T*

*C*
