# Supplementary material for: Association Mapping of Insecticide Resistance in Wild Anopheles gambiae Populations: Major Variants Identified in a Low-Linkage Disequilbrium Genome
Source: PLoS One. 2010 Oct 1;5(10):e13140. doi: 10.1371/journal.pone.0013140 (PMC2956759; doi:10.1371/journal.pone.0013140)
Supplement: Table S2 — Clustering and 2La karyotypes. Relationship been multilocus genotype clusters and 2La karyotypes in each major subpopulation. (0.04 MB DOC) [file pone.0013140.s004.doc]

Table S2. Relationship between multilocus genotype clusters (nomenclature as in Figure 2) and 2La karyotypes, for a portion of the samples diagnosed by PCR [31], for the three major subpopulations in the dataset. Probabilities for rejection fo Hardy-Weinberg (H-W) proportions are shown.

|  |  | karyotype (PCR diagnostic) | | |  | |  |
| --- | --- | --- | --- | --- | --- | --- | --- |
|  | cluster | 2La | 2La/2L+^a^ | 2L+a/2L+^a^ | H-W prob | | |
|  |  |  |  |  |  |  | |
| Cam M | c1-90 | 6 | 1 | 0 |  |  | |
|  | c2-330 | 12 | 3 | 0 |  |  | |
|  | c3-253 | 12 | 2 | 0 | 1.00 |  | |
| Cam S | c4-15 | 0 | 12 | 1 |  |  | |
|  | c5-47 | 23 | 0 | 0 | 1.00 |  | |
| Gha S | c2-377 | 0 | 36 | 0 |  |  | |
|  | c6-116 | 16 | 0 | 0 |  |  | |
|  | c7-233 | 0 | 0 | 26 | 0.88 |  | |
